# Gold nanoparticle-enhanced X-ray microtomography of the rodent reveals region-specific cerebrospinal fluid circulation in the brain

**DOI:** 10.1038/s41467-023-36083-1

**Published:** 2023-01-27

**Authors:** Shelei Pan, Peter H. Yang, Dakota DeFreitas, Sruthi Ramagiri, Peter O. Bayguinov, Carl D. Hacker, Abraham Z. Snyder, Jackson Wilborn, Hengbo Huang, Gretchen M. Koller, Dhvanii K. Raval, Grace L. Halupnik, Sanja Sviben, Samuel Achilefu, Rui Tang, Gabriel Haller, James D. Quirk, James A. J. Fitzpatrick, Prabagaran Esakky, Jennifer M. Strahle

**Affiliations:** 1 Department of Neurosurgery, Washington University School of Medicine, Washington University in St. Louis, St. Louis, MO 63110 USA; 2 Washington University Center for Cellular Imaging, Washington University School of Medicine, Washington University in St. Louis, St. Louis, MO 63110 USA; 3 Department of Radiology, Washington University School of Medicine, Washington University in St. Louis, St. Louis, MO 63110 USA; 4 Department of Neurology, Washington University School of Medicine, Washington University in St. Louis, St. Louis, MO 63110 USA; 5 Department of Biomedical Engineering, Washington University in St. Louis, St. Louis, MO 63110 USA; 6 Department of Biomedical Engineering, UT Southwestern Medical Center, Dallas, TX 75390 USA; 7 Department of Genetics, Washington University School of Medicine, Washington University in St. Louis, St. Louis, MO 63110 USA; 8 Department of Neuroscience, Washington University School of Medicine, Washington University in St. Louis, St. Louis, MO 63110 USA; 9 Department of Cell Biology and Physiology, Washington University School of Medicine, Washington University in St. Louis, St. Louis, MO 63110 USA; 10 Department of Orthopedic Surgery, Washington University School of Medicine, Washington University in St. Louis, St. Louis, MO 63110 USA; 11 Department of Pediatrics, Washington University School of Medicine, Washington University in St. Louis, St. Louis, MO 63110 USA

**Keywords:** Development of the nervous system, Diseases of the nervous system

## Abstract

Cerebrospinal fluid (CSF) is essential for the development and function of the central nervous system (CNS). However, the brain and its interstitium have largely been thought of as a single entity through which CSF circulates, and it is not known whether specific cell populations within the CNS preferentially interact with the CSF. Here, we develop a technique for CSF tracking, gold nanoparticle-enhanced X-ray microtomography, to achieve micrometer-scale resolution visualization of CSF circulation patterns during development. Using this method and subsequent histological analysis in rodents, we identify previously uncharacterized CSF pathways from the subarachnoid space (particularly the basal cisterns) that mediate CSF-parenchymal interactions involving 24 functional-anatomic cell groupings in the brain and spinal cord. CSF distribution to these areas is largely restricted to early development and is altered in posthemorrhagic hydrocephalus. Our study also presents particle size-dependent CSF circulation patterns through the CNS including interaction between neurons and small CSF tracers, but not large CSF tracers. These findings have implications for understanding the biological basis of normal brain development and the pathogenesis of a broad range of disease states, including hydrocephalus.

## Abstract (web summary)

Cerebrospinal fluid (CSF) is essential for brain development. Here, the authors use gold nanoparticle enhanced X-ray microtomography to map CSF distribution in post-natal rodents and describe particle size-dependent CSF pathways that transport CSF into the brain parenchyma of rodents.

## Introduction

Postnatal brain development involves dramatic changes in the cells and structure of the brain and the establishment of interconnected signaling pathways with newborn neurons arising from the subventricular zone (SVZ) and hippocampus^1^. These neural precursor cell populations are adjacent to the cerebrospinal fluid (CSF) spaces and are thought to be modulated by CSF constituents that change during development^2–6^. CSF has been shown to promote the function and survival of glia and neurons in vitro and may have a role in modulating neuronal activity^5,7–12^.

CSF circulation has recently received renewed interest, in part due to the newly described pathways for fluid movement via the glymphatic and lymphatic pathways and their putative role in neurodegenerative diseases such as Alzheimer’s Disease and multiple sclerosis^13–15^. These routes for CSF movement have primarily been studied in adult and senescent animals in relation to waste removal. The understanding of CSF as a source of nutrient and growth factor delivery in the developing brain is still evolving.

In fact, our current understanding of CSF circulation is not representative of the conditions in developing animals, calling into question its relevance to some of the most common CSF disorders of childhood, such as hydrocephalus. For example, the historically implicated structures for CSF handling along the superior sagittal sinus, arachnoid villi, and granulations, are not fully developed in humans until two years of age and are not present in a number of species, including rodents^16–18^. In addition, while sparse meningeal lymphatics have been observed in mice at birth around the foramen magnum and pterygopalatine artery^19^, most functional meningeal lymphatic connections take three to four weeks to fully develop postnatally^19,20^. Finally, glymphatic CSF handling has primarily been studied in adult animal models (with a few exceptions^21–24^), and most studies have focused on the superficial cortical surface adjacent to the middle cerebral artery (MCA) as it can be visualized with two-photon live imaging^13^. CSF clearance via the Na^+^/K^+^/Cl^−^ cotransporter 1 (NKCC1) during postnatal development was recently reported^25^, however, this and other components of putative CSF movement, including lymphatic CSF outflow, glymphatic fluid and solute handling, perineural, perivenous, and spinal subarachnoid space (SAS) CSF handling, have been studied largely in isolation from one another^15,26–28^. Few, if any, prior analyses of CSF dynamics have simultaneously been able to examine the cerebrum, cerebellum, and spinal cord due to imaging constraints, a limitation that hampers our ability to understand global CSF circulation throughout the central nervous system (CNS).

Special considerations for the study of CSF in the developing brain include the concern that disruption of CSF dynamics interrupts normal brain development^29–32^. More fundamental is the enigma surrounding the directionality of the relationship between CSF accumulation and neurodevelopmental aberrations^29^. Specifically, CSF circulation has largely been thought of in the context of secretion and drainage without consideration for how CSF (and its solutes) interfaces with specific populations of developing neurons and glia in the CNS. In this present study, we address how CSF interacts with the brain and spinal cord by utilizing a CSF imaging method, gold nanoparticle-enhanced X-ray microtomography (AuNP-XRM), for high-resolution 2-D and 3-D imaging of CSF pathways through the entire CNS of neonatal rats with histologic confirmation^33–35^. We present a comprehensive descriptive map of differential CSF influx to intraparenchymal functional-anatomic targets. Small CSF tracers travel via previously uncharacterized influx pathways from the basal cisterns to deep functional-anatomic cell groupings, and from the SAS to the cerebral and cerebellar cortices. Large CSF tracers enter the extracellular space within the hippocampus and septal area via transventricular, transependymal, and transleptomeningeal routes, contrasting the small CSF tracer targets, which are primarily neurons and functional-anatomic cell populations. CSF delivery to these regions is altered in a model of known CSF pathology^36,37^, intraventricular hemorrhage-post-hemorrhagic hydrocephalus (IVH-PHH), with implications for mechanisms of brain injury and impaired brain development in PHH.

## Results

### CSF circulation imaging using AuNP-XRM

**Fig. 1:**
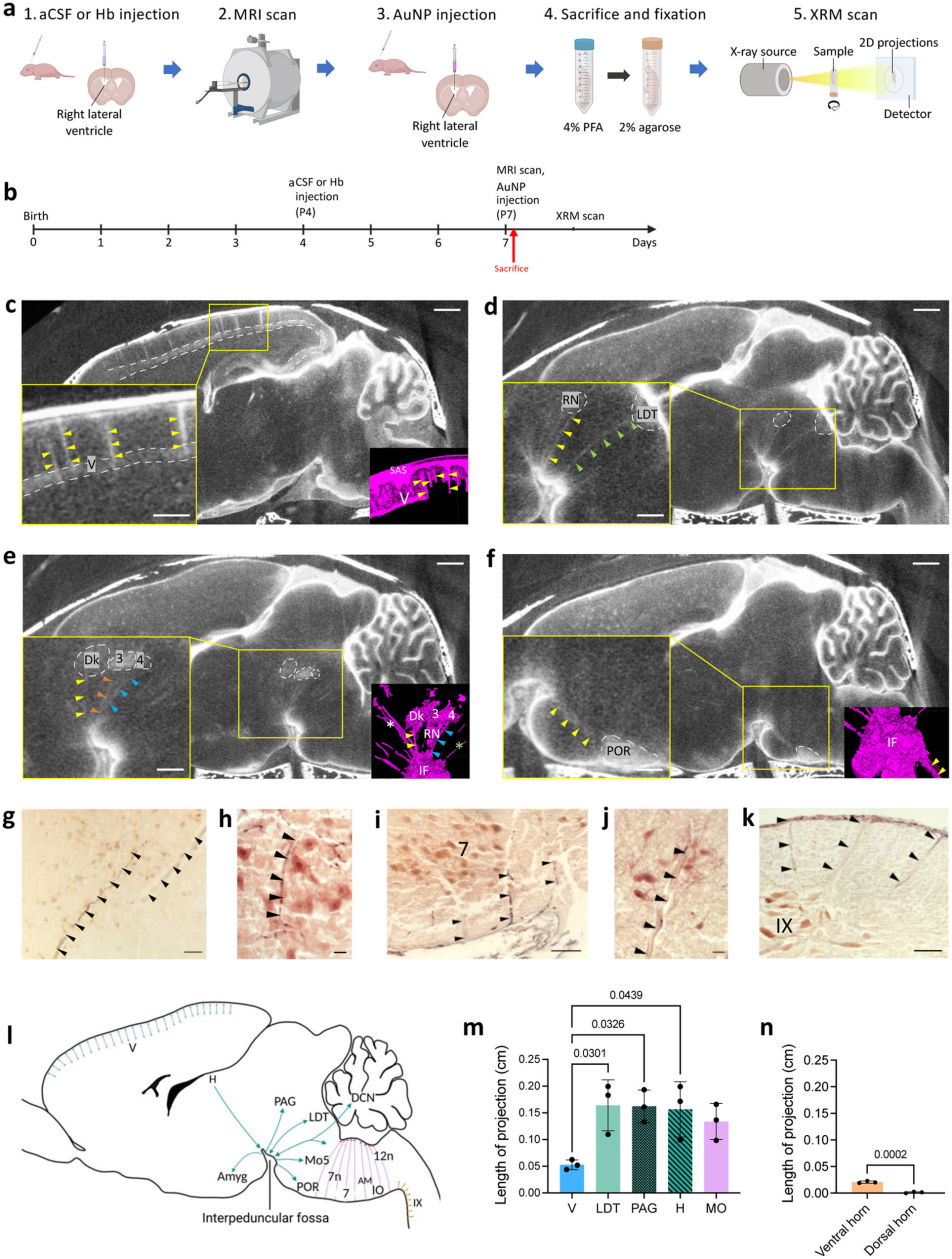
CSF pathways target intraparenchymal cell populations throughout the forebrain, midbrain, hindbrain, and spinal cord. Workflow (**a**) and timeline (**b**) for 1.9 nm gold nanoparticle enhanced X-ray microtomography (AuNP-XRM). **c**–**f** Sagittal XRM demonstrates CSF pathways arising from the subarachnoid space (SAS) to deliver 1.9 nm AuNPs to layer V of the cerebral cortex (yellow arrows, V) (**c**). There were also prominent basal CSF pathways arising from the interpeduncular fossa (IF) and basal cisterns to deliver AuNPs to the red nucleus (yellow arrows, RN) and laterodorsal tegmentum (green arrows, LDT) (**d**); the nucleus of Darkschewitsch (yellow arrows, DK), oculomotor nucleus (orange arrows, 3), and trochlear nucleus (blue arrows, 4) (**e**); and the periolivary region (yellow arrows, POR) (**f**). Higher magnification insets (bottom left) and 3D segmentations (bottom right) provide alternative visual representations of these CSF pathways. XRM scalebar = 1 mm, inset scalebar = 500 µm. **g–k** Histology showing CSF pathways into the hypothalamus (**g**), facial nerve nucleus (**h**, **i**), nucleus ambiguus (**j**), and lamina IX of the spinal cord (**k**). Black arrowheads indicate AuNP CSF pathways. **g** Scalebar = 50 µm, **h** scalebar = 25 µm, i scalebar = 100 µm, **j** scalebar = 25 µm, **k** scalebar = 100 µm. **l** Schematic representation of CSF pathways and a subset of their intraparenchymal targets identified with AuNP-XRM. Abbreviations: V, layer V of the cortex; H, habenula; Amyg, amygdala; PAG, periaqueductal gray (including oculomotor nucleus); LDT, laterodorsal tegmentum; Mo5, motor nucleus of the trigeminal nerve; POR, periolivary nucleus; 7, 7n, facial nucleus; AM, nucleus ambiguus; 12n, hypoglossal nucleus; IO, inferior olivary nucleus; DCN, deep cerebellar nuclei, IX, lamina IX of the spinal cord. **m** 1.9 nm AuNP influx depth into the brain parenchyma along basal and cortical CSF pathways. Data are mean ± S.D., *n* = 3 rodents, One-way ANOVA with post-hoc Tukey. Abbreviations: V, layer V of the cortex; LDT, laterodorsal tegmentum; PAG, periaqueductal gray, H, habenula; MO, medulla oblongata. **n** 1.9 nm AuNP influx depth into the spinal cord parenchyma along ventral and dorsal CSF pathways. Data are mean ± S.D., *n* = 3 rodents, unpaired two tailed t-test. Source data are provided in Supplementary Information. Figure 1a, l were created with BioRender.com.

To image all pathways for CSF movement within the CNS, we utilized high resolution XRM with AuNPs as a CSF tracer (Fig 1a)^33–35^. Polyethylene glycol (PEG)-coated AuNPs constituted in artificial CSF (aCSF) were injected into the CSF spaces as a contrast agent and CSF tracer. Following injection, samples were prepared for XRM, as shown in Figs. 1a, b (also see “Methods”). The use of this method resulted in high resolution (14.7 μm/pixel) images of the entire brain and spinal cord in which the CSF spaces and parenchyma including discrete nuclei (cell groupings), tracts, and cranial nerves could be clearly visualized. The AuNPs have high X-ray attenuation, low negative zeta potentials (−1.3 ± 0.4 mV for 1.9 nm AuNPs and −1.6 ± 0.8 mV for 15 nm AuNPs), and average hydrodynamic diameters of 3.79 ± 0.16 and 35.91 ± 0.62 nm for 1.9 and 15 nm AuNPs, respectively.

### Targeted CSF delivery to functional intraparenchymal nuclei and cell groupings

Unlike prior studies in adult rodents in which CSF tracers were observed in dorsal and cortical structures with emphasis on localization around the perivascular spaces of major and penetrating arteries^13,38,39^, 1.9 nm AuNP-XRM and corresponding histology revealed a robust pattern of CSF pathways entering the brain parenchyma from the interpeduncular and pontine cisterns as early as 10 minutes after intraventricular 1.9 nm AuNP injection (Fig. 1d–j). 1.9 nm AuNPs also traveled along CSF pathways that extended from the SAS over the dorsal convexities into layers III and V of the cortex (Fig. 1c) and spinal SAS into the ventral horn of the spinal cord (Fig. 1k).

Multiple CSF pathways arose from the dorsal convexity SAS and interpeduncular and pontine cisterns, each with a distinct trajectory into the parenchyma (Fig. 1l, m). CSF pathways arising from the interpeduncular cistern penetrated deeper compared to those in the dorsal convexities (Fig. 1m). Similarly, CSF pathways into the ventral horn of the spinal cord penetrated deeper into the spinal cord parenchyma compared to those in the dorsal horn (Fig. 1n).

To rationalize these differences, we hypothesized that the CSF pathways coordinate a previously unidentified, targeted CSF circulation within the CNS that delivers CSF to specific regions of the brain and spinal cord. Supporting this hypothesis, we identified focal, intense regions of 1.9 nm AuNPs at the terminations of the CSF pathways that correspond to discrete anatomical and functional cell groupings in the forebrain, midbrain, brainstem, cerebellum, and spinal cord^40,41^. CSF pathways arising from the interpeduncular cistern traveled through the midbrain and pons and terminated in the red nucleus and laterodorsal tegmentum (Fig. 1d); oculomotor nucleus, nucleus of Darkschewitsch, and trochlear nucleus (Fig. 1e); periolivary region of the superior olivary complex (Fig. 1f); facial nerve nucleus (Fig. 1h, i); and nucleus ambiguus (Fig. 1j). Cortical SAS CSF pathways terminated in layers III and V of the cerebral cortex (Fig. 1c), and spinal SAS CSF pathways terminated in lamina IX in the ventral horn (Fig. 1k).

**Fig. 2:**
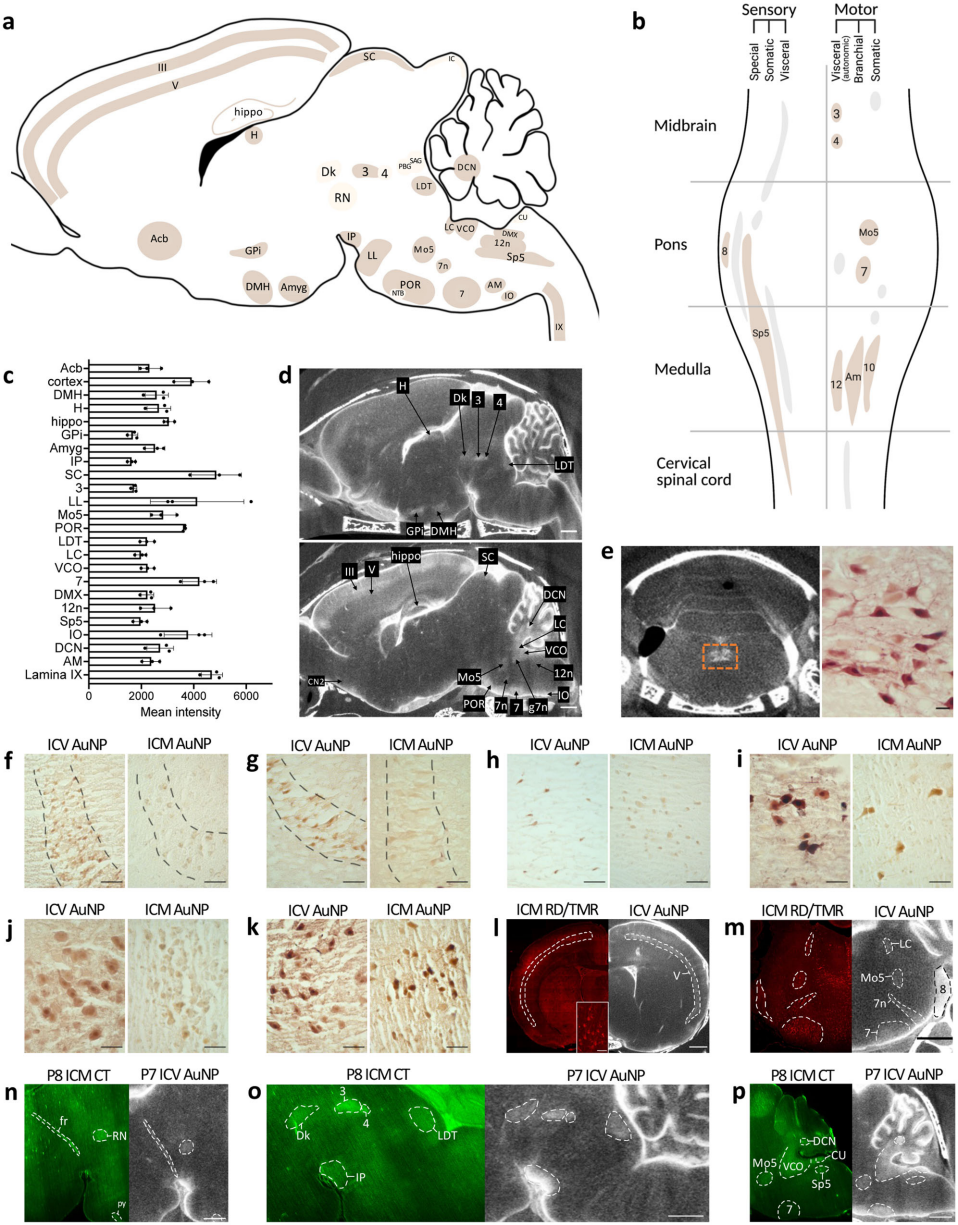
Identification of 24 functional-anatomic cell groupings that preferentially interact with CSF. **a** Schematic of functional-anatomic regions in which 1.9 nm gold nanoparticles (AuNPs) interact with cell populations 10 min post-intraventricular injection in P7 rodents. 24 areas identified in all rodents (brown) include the nucleus accumbens (Acb), layers III and V of the cortex (III, V), dorsal medial hypothalamus (DMH), habenula (H), hippocampus (hippo), globus pallidus internus (GPi), amygdala (amyg), interpeduncular nucleus (IP), superior colliculus (SC), oculomotor nucleus (3), lateral lemniscus (LL), motor trigeminal nucleus (Mo5), periolivary region (POR), laterodorsal tegmentum (LDT), locus coeruleus (LC), ventral cochlear nucleus (VCO), facial nucleus (7, 7n), dorsal nucleus of the vagus (DMX), hypoglossal nucleus (12n), spinal trigeminal nucleus (Sp5), inferior olive (IO), deep cerebellar nuclei (DCN), nucleus ambiguus (AM), and lamina IX (IX). 1.9 nm AuNPs were also identified in the nucleus of Darkschewitsch (Dk), red nucleus (RN), trochlear nucleus (4), and nucleus of trapezoid body (NTB) in one rodent (light brown), and the nucleus sagulum (SAG), external cuneate nucleus (CU), inferior colliculus (IC), pontine reticular nucleus, and parabigeminal nucleus (PBG) in two (light brown). **b** Schematic showing cranial nerve nuclei with 1.9 nm AuNP uptake (brown) are primarily motor. **c** 1.9 nm AuNP mean intensity in the 24 regions. Data are mean ± S.D., *n* = 3 rodents. **d** X-ray microtomography (XRM) images showing 1.9 nm AuNP distribution. scalebars = 1 mm. **e** Coronal XRM (left) and histology (right) showing 1.9 nm AuNP distribution in the 12n (orange box). XRM scalebar = 1 mm, histology scalebar = 25 µm. **f–k** Histology showing similar 1.9 nm AuNP distribution patterns in the cortex (**f**), hippocampus (**g**), hypothalamus (**h**), Mo5 (**i**), 7n (**j**), and 12n (**k**) after intraventricular (ICV) and intra cisterna magna (ICM) injection in P7 rats. scalebars = 50 µm. **l**, **m** Confocal microscopy and XRM showing similar distribution patterns in the cortex, LC, Mo5, and 7n following ICM injection of Red-Dextran/tetramethylrhodamine (RD/TMR) and ICV 1.9 nm AuNP. scalebars = 1 mm. **n**–**p** Fluorescence microscopy and XRM showing similar distribution patterns in the fasciculus retroflexus (fr), RN, pyramidal tract (py), IP, oculomotor complex, 4, LDT, Mo5, 7, VCO, Sp5, DCN, and CU between a P8 mouse injected with ICM CellTracker (CT) and a P7 rat injected with ICV 1.9 nm AuNPs. scalebars = 1 mm. Source data are provided in Supplementary Information. Figure 2a, b were created with BioRender.com.

We quantified the 1.9 nm AuNP-XRM signal intensity in each functional-anatomic region against the surrounding background and identified 24 nuclei and cell groupings with a significant increase in 1.9 nm AuNPs across three rodents (Fig. 2a–e, Supplementary Fig. 1, and Supplementary Table 1). There were also four nuclei (red nucleus, nucleus of Darkschewitsch, trochlear nucleus, nucleus of the trapezoid body) with increased 1.9 nm AuNPs in one rodent, and five nuclei and structures (nucleus sagulum, parabigeminal nucleus, inferior colliculus, pontine reticular nucleus, and external cuneate nucleus) with increased 1.9 nm AuNPs in two rodents. In addition, we observed 1.9-nm AuNPs in the fasciculus retroflexus and pyramidal tracts. There was also widespread distribution in the molecular layer of the cerebellum in all three rodents (Supplementary Fig. 2). In the spinal cord, 1.9 nm AuNPs were identified in lamina IX of all rodents, however, there were no AuNPs in the dorsal horn (Supplementary Fig. 1x); this mirrors the absence of dorsal spinal cord influx pathways observed on AuNP-XRM (Fig. 1n).

To confirm location within the anatomic regions identified on 1.9 nm AuNP-XRM, the brain and spinal cord were histologically sectioned without any additional markers or counterstains. 1.9 nm AuNPs, which appear brown under light microscopy, were observed interacting with cells in regions corresponding to the same functional-anatomic locations seen on AuNP-XRM (Supplementary Figure 1). In addition to the targeted distribution to the 24 functional-anatomic cell groupings via the CSF pathways that we identified, there was a diffuse, global distribution of 1.9 nm AuNPs to the parenchyma at 10 min. The corpus callosum (anterior forceps, body, genu, and splenium) was the only brain region that did not have 1.9 nm AuNP distribution or uptake, which highlights preferential CSF influx to the gray matter.

### CSF pathways are preserved across tracer types and CSF administration modalities

We used five methods to validate our findings. First, one 1.9 nm AuNP-XRM image was registered to the Duke Wistar rat atlas^42,43^ using 4dfp tools (http://4dfp.readthedocs.io); the skull was stripped and regions of intraparenchymal CSF were semi-automatically segmented to reveal the same patterns of CSF distribution as were manually quantified, suggesting our findings are not the result of human artifact (Fig. 1c–f bottom right insets).

Second, to evaluate whether the observed CSF distribution patterns were independent of the physical and chemical characteristics of the 1.9 nm AuNPs and to confirm similar distributions in vivo, we acquired in vivo contrast-enhanced T1 MRIs and observed dark regions indicating very high focal concentrations of Gd-DOTA (gadoterate meglumine) in functional-anatomic cell groupings similar to those seen with AuNP-XRM (Supplementary Fig. 3). We were not able to discern all 24 functional-anatomic cell groupings identified on XRM due to the lower-resolution nature of the Gd-DOTA-enhanced T1 MRI (AuNP-XRM pixel size = 14.73 × 14.73 × 14.73 µm^3^, Gd-DOTA-enhanced T1 MRI voxel size = 58.59375 × 58.59375 × 1000.0000 µm^3^). These similar patterns suggest that the 1.9 nm AuNP distribution is not an artifact of the ex vivo nature of XRM or specific to AuNPs but rather generalizable to small CSF tracers.

Third, to address the effect of potential acute brain injury from intraventricular injection on CSF distribution, we injected 1.9 nm AuNPs into the cisterna magna (ICM) of P7 rats. We found similar AuNP distribution patterns throughout all but three functional-anatomic cell groupings evaluated within the brain 10 min post-injection to that observed with intraventricular injection (Fig. 2f–k and Supplementary Table 1). While we were able to identify many of the same regions, it qualitatively appeared that there was less AuNP delivery to these functional-anatomic cell groupings following ICM injection compared to intraventricular delivery, particularly to more anterodorsal structures like the cortex. We observed comparable patterns following ICM injection of 3 kDa Red-Dextran/Tetramethylrhodamine (RD/TMR) under equivalent conditions (Fig. 2l, m and Supplementary Table 1). Similar to previous studies examining differences in spinal CSF tracer distribution following intraventricular vs. ICM injection^44^, we did not see substantial amounts of tracer in the thoracic or lumbar spine following injection into the cisterna magna.

Fourth, cresyl violet counterstaining confirmed that only a subpopulation of cells (i.e., not all cells) within each functional-anatomic cell grouping contained gold. This ranged from 12 to 67% of all cells, suggesting a unique CSF uptake function for cells with vs. without gold (Supplementary Fig. 4), and confirming that the CSF uptake within these cell groupings was not uniform across all cells. Six of the functional-anatomic cell groupings from ICM RD/TMR-injected rodents were evaluated for NeuN expression to confirm neuronal cell type in conjunction with morphology (Supplementary Fig. 5). All RD/TMR+ cells colocalized with NeuN in 4/6 of the areas evaluated (hippocampus, interpeduncular nucleus, motor nucleus of the trigeminal nerve, hypoglossal nucleus), suggesting the cells interacting with CSF tracers in these regions are neurons (Supplementary Fig. 5a, b, g, h, i, j, k, l). In the two other regions (globus pallidus and cortex), 70–88% of RD/TMR+ cells were also NeuN+, suggesting the majority but not all of the cells that interact with CSF in these regions are neurons (Supplementary Fig. 5c–f). It is also possible the CSF interacts with a subset of non-neuronal cells in the other 18 regions that were not evaluated with NeuN.

Finally, to evaluate cross-species preference of small CSF tracers for the 24 identified functional-anatomic cell groupings, we performed ICM injection of CellTracker Green fluorescent tracer in P8 mice and found similar patterns of tracer distribution to both intraventricular and ICM injection of 1.9 nm AuNPs in P7 rats (Fig. 2n–p and Supplementary Table 1). CellTracker injections in P21 mice revealed that in contrast to the patterns observed at P8 there was no tracer uptake in the cortex, hippocampus, or a subset of brainstem nuclei at P21, suggesting that the CSF circulation patterns within these areas may be age-dependent (Supplementary Fig. 6 and Supplementary Table 1). Furthermore, intraventricular injection of 70 kDa RD into the ventricles of 4 days post-fertilization (dpf) zebrafish resulted in RD distribution throughout regions known to contain high gray matter density (referencing the Max Planck Zebrafish Brain (mapZebrain) atlas), similar to the patterns observed with all of the previously mentioned experimental parameters (Supplementary Fig. 7).

### CSF distribution mirrors choline acetyltransferase expression in the brain

Given the diverse anatomic locations of these 24 cell groupings, we hypothesized that the regions receiving targeted CSF influx are correlated with the expression of genes, neurotransmission, or function. Notably, seven of nine cranial nerve nuclei with 1.9 nm AuNP CSF tracer distribution were motor-derived cranial nerve nuclei (Fig. 2b). Furthermore, the other 15 non-cranial nerve functional-anatomic cell groupings had roles in motor, learning, and memory functions. A systematic review for expression patterns similar to the localization of the CSF tracers was performed using the Gene Expression Nervous System Atlas (GENSAT)^45^ and the Allen Mouse Brain Atlas Anatomic Gene Expression Atlas (AGEA)^46^ of the developing brain and revealed similar patterns to that of the enzyme choline acetyltransferase (ChAT) (Supplementary Fig. 8 and Supplementary Tables 2, 3). Co-localization of tissue sections for ChAT and AuNPs was not possible as AuNPs block transmitted light. Therefore, histologic sections from a separate cohort of rats without AuNP injection were stained for ChAT. All but 5 of the 24 functional-anatomic cell groupings had ChAT immunofluorescence in the same anatomic region as the 1.9 nm AuNP localization. (Supplementary Fig. 8). Furthermore, ChAT immunofluorescence staining in a subset of regions on tissue sections from rats injected with ICM Red-Dextran/TMR revealed co-localization of ChAT on cells with Red-Dextran uptake. All cell bodies with RD/TMR uptake within the globus pallidus, cortex, and motor nucleus of the trigeminal nerve were also ChAT+, however not all ChAT+ cells took up RD/TMR (Supplementary Fig. 9). Cross reference with an atlas (GENSAT) and previously published data supported parallel ChAT expression to CSF circulation patterns (Supplementary Table 2 and Supplementary Table 3)^45^.

### Targeted CSF distribution is altered in neonatal posthemorrhagic hydrocephalus

**Fig. 3:**
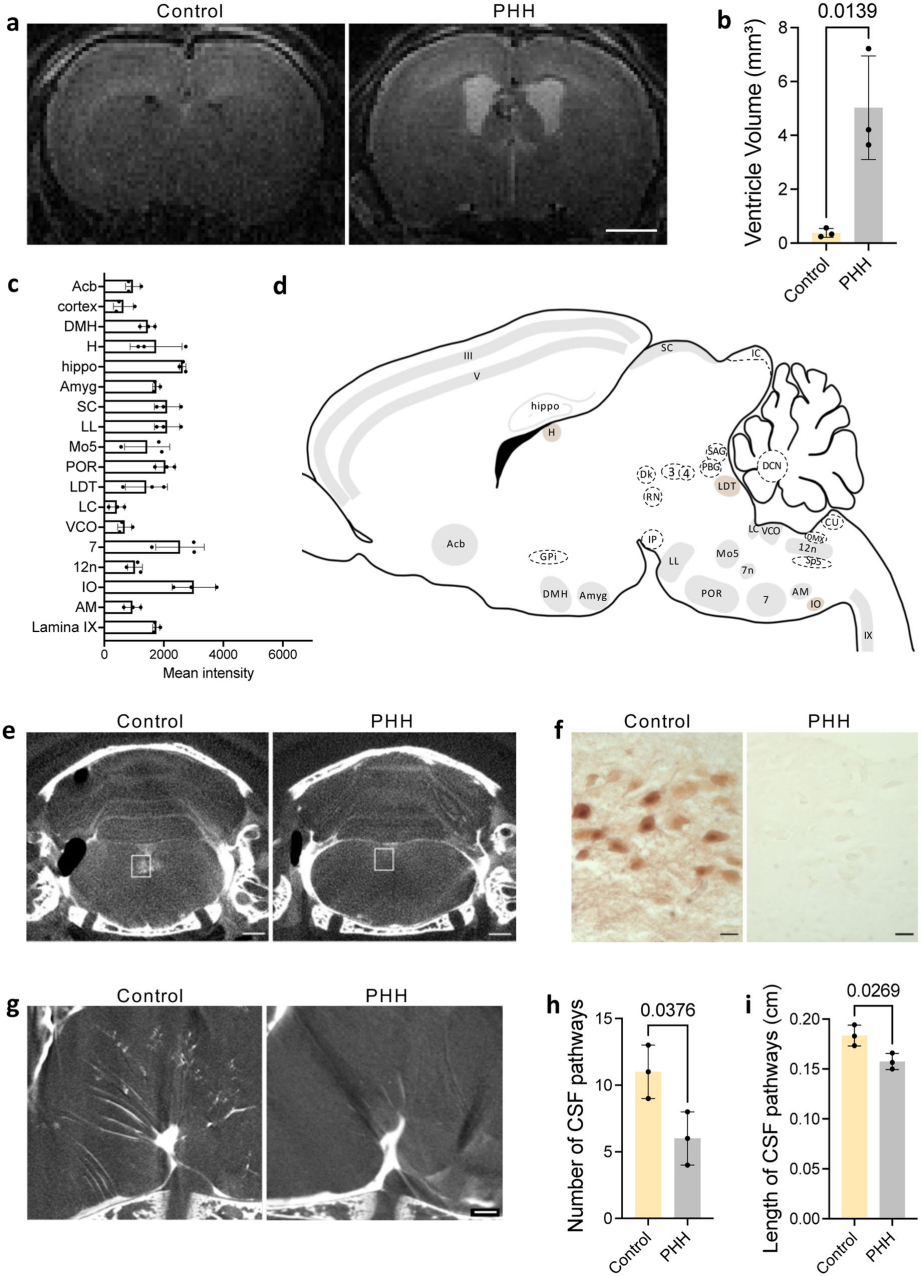
CSF distribution to functional-anatomic cell groupings is altered in post hemorrhagic hydrocephalus. **a** T2-weighted MRI showing ventriculomegaly 72 h after hemoglobin injection into the right lateral ventricle of P4 rodents to induce intraventricular hemorrhage-posthemorrhagic hydrocephalus (IVH-PHH). scalebar = 1 mm. **b** Lateral ventricle volume was significantly increased in IVH-PHH rodents compared to the aCSF controls. Data are mean ± S.D., *n* = 3 rodents, unpaired two-tailed t-test. **c** 1.9 nm gold nanoparticle (AuNP) mean intensity in 18 functional-anatomic cell groupings in IVH-PHH rodents 10 minutes after intraventricular injection at P7. Data are mean ± S.D., *n* = 3 rodents. **d** Schematic representation of functional-anatomic cell groupings with significantly decreased 1.9 nm AuNP intensity in IVH-PHH rodents (compared to aCSF controls) (gray). Cell groupings with no significant change in AuNP intensity are shown in brown and those that were not able to be consistently identified after PHH are shown in white with a dotted outline. Abbreviations: nucleus accumbens (Acb), layers III and V of the cortex (III, V), dorsal medial hypothalamus (DMH), habenula (H), hippocampus (hippo), globus pallidus internus (GPi), amygdala (amyg), interpeduncular nucleus (IP), superior colliculus (SC), oculomotor nucleus (3), lateral lemniscus (LL), motor trigeminal nucleus (Mo5), periolivary region of the superior olivary complex (POR), laterodorsal tegmentum (LDT), locus coeruleus (LC), ventral cochlear nucleus (VCO), facial nerve nucleus (7, 7n), dorsal nucleus of the vagus nerve (DMX), hypoglossal nucleus (12n), spinal trigeminal nucleus (Sp5), inferior olivary nucleus (IO), deep cerebellar nuclei (DCN), nucleus ambiguus (AM), and lamina IX of the spinal cord (IX). **e–f** Coronal X-ray microtomography (XRM) (**e**) and histology (**f**) images showing AuNP distribution in the hypoglossal nucleus (12n) (white box) in aCSF control and IVH-PHH rodents. **e** Scalebar = 1 mm, **f** scalebar = 25 µm. **g–i** XRM (**g**) and quantifications (**h**, **i**) showing changes in the number and length of basal CSF pathways in IVH-PHH rodents. **g** Scalebar = 100 µm. Data are mean ± S.D., *n* = 3 rodents, unpaired two-tailed t-test. Coronal XRM, histology, and quantifications of the other 17 regions (*n* = 3 rodents per condition) are shown in Supplementary Fig. 10. Source data are provided in Supplementary Information. Figure 3d was created with BioRender.com.

Posthemorrhagic hydrocephalus (PHH) after preterm intraventricular hemorrhage (IVH) is associated with the worst neurocognitive outcomes in infants born preterm in the United States^47^. The etiology for PHH is not known and studies of individual mechanisms have not resulted in effective therapeutic targets for the prevention of PHH or associated brain injury^48^. Our findings of targeted CSF delivery to 24 functional-anatomic cell groupings suggest that the CSF pathways from the cisterns and SAS may have a functional role in brain development. Therefore, we hypothesized that altering CSF flow by inducing hydrocephalus would lead to changes in the targeted CSF pathways and 1.9 nm AuNP delivery to the 24 regions. To test this hypothesis, we induced IVH-PHH with hemoglobin (Hb) injection into the right lateral ventricle of P4 rodents (Figs. 1a, 3a, b)^36,37,49^. Ventricle volume was quantified using T2-weighted MR images acquired 72 hours post-injection and demonstrated a significant enlargement in ventricle size in PHH (5.03 ± 1.38 mm^3^) compared to aCSF controls (0.38 ± 0.14 mm^3^) (Fig. 3a, b).

We found that IVH-PHH was associated with significantly decreased 1.9 nm AuNP delivery to 15 of the 24 identified regions. (Fig. 3c, d and Supplementary Fig. 10a–c, e–j, l–o, q, r). There were also non-significant differences in the 1.9 nm AuNP distribution in 3 of 24 regions (Supplementary Fig. 10d, k, p). 1.9 nm AuNP distribution in the aCSF control and PHH conditions in the remaining 6 regions (globus pallidus internus, interpeduncular nucleus, oculomotor nucleus, dorsal motor nucleus of the vagus nerve, spinal trigeminal nucleus, deep cerebellar nuclei) were not compared because the regions were not easily identifiable in the PHH group. Furthermore, we observed significantly fewer distinct CSF pathways from the interpeduncular cistern in PHH rodents (Fig. 3g, h), with the remaining pathways appearing significantly shorter (Fig. 3i).

### Transventricular, transependymal, and transleptomeningeal circulation of large CSF tracers

**Fig. 4:**
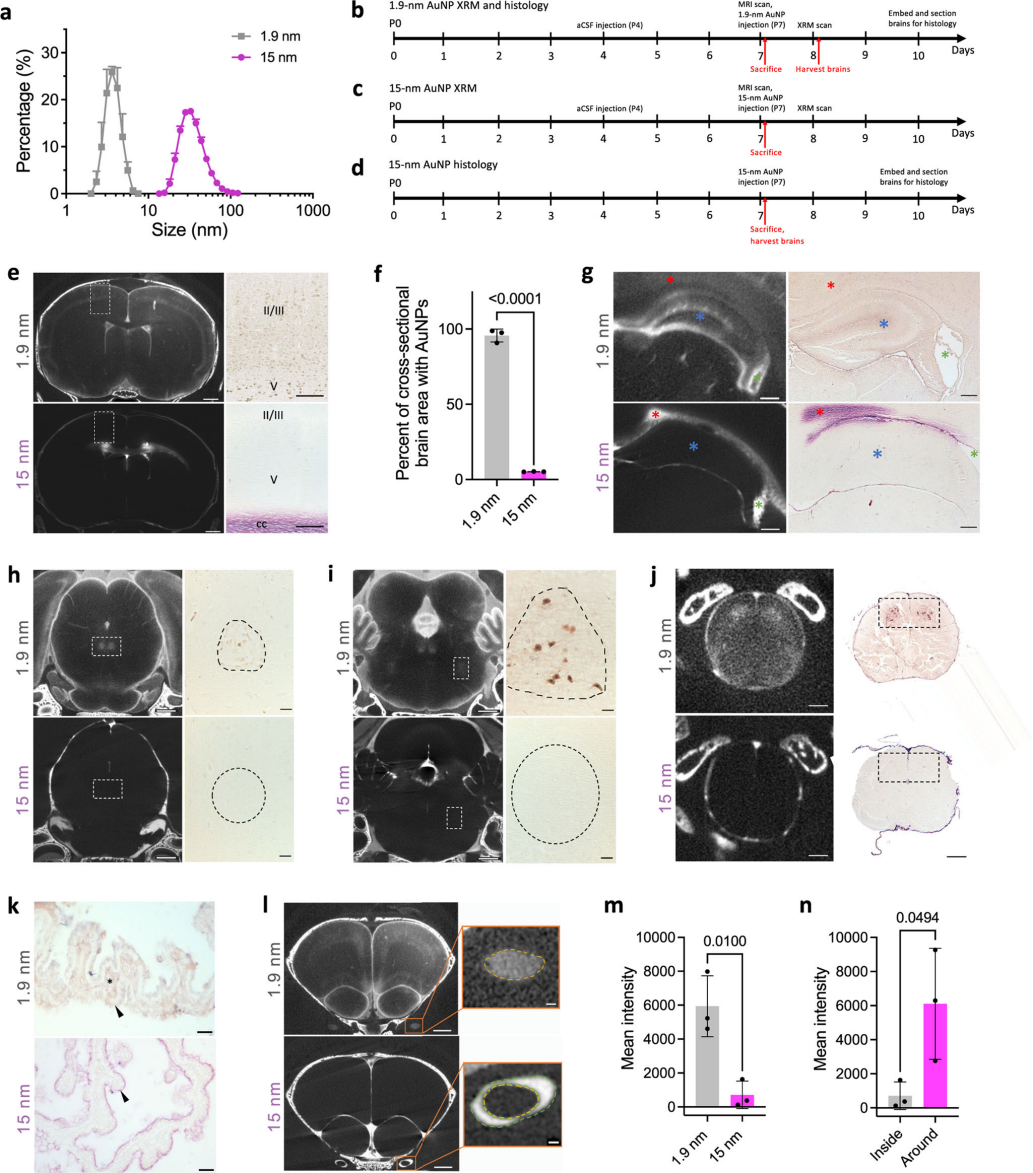
Size-dependent CSF tracer distribution in the parenchyma, choroid plexus, and optic nerve. **a** Hydrodynamic diameters of 1.9 and 15 nm gold nanoparticles (AuNPs) are 3.79 and 35.91 nm, respectively. Data are representative of *n* = 32 15 nm AuNPs and *n* = 30 1.9 nm AuNPs. **b–d** Experimental timelines for 1.9 nm AuNP X-ray microtomography (XRM) and histology (**b**), 15 nm AuNP-XRM (**c**), and 15 nm histology (**d**). 1.9 nm AuNP-XRM followed the same workflow as described in Fig. 1a, b. **e** XRM and histology of 1.9 and 15 nm AuNP cortical distribution 10 min after intraventricular injection in P7 rodents. 15 nm AuNP distribution was primarily extracellular, while 1.9 nm AuNPs interacted with cells. XRM scalebar = 1 mm, histology scalebar = 100 µm. **f** 1.9 nm AuNPs cover more cross-sectional brain area than 15 nm AuNPs. Data are mean ± S.D., *n* = 3 rodents, unpaired two-tailed t-test. **g** XRM and histology showing 15 nm AuNP movement into the corpus callosum (red asterisk) but not the hippocampus (blue asterisk) 10 min post-injection into the lateral ventricles (LV) (green asterisk). XRM scalebars = 250 µm, histology scalebars = 250 µm. **h–j** XRM and histology of 1.9 and 15 nm AuNP distribution in the midbrain and oculomotor nucleus (**h**), pons and trigeminal motor nucleus (**i**), and spinal cord (**j**). **h**, **i** XRM scalebars = 1 mm, histology scalebars = 50 µm; **j** XRM scalebars = 250 µm, histology scalebars = 250 µm. **k** Histology of 1.9 nm (top) and 15 nm (bottom) AuNP distribution in the right LV choroid plexus 10 min after AuNP injection showing apical (15 nm, black arrowhead) vs. apical and basolateral (1.9 nm, black arrowhead and asterisk respectively) choroid plexus coverage. scalebars = 25 µm. **l**–**n** XRM of 1.9 nm (top) and 15 nm (bottom) AuNP distribution in and around the optic nerve (**l**) and mean intensity of 1.9 and 15 nm AuNPs within the optic nerve (**m**) 10 min after 1.9 nm AuNP injection and 4 h after 15 nm AuNP injection. 15 nm AuNPs were primarily located around (vs. within) the optic nerve (**n**). **l** scalebar = 1 mm, inset scalebar = 100 µm. **m**, **n** Data are mean ± S.D., *n* = 3 rodents, unpaired two-tailed t-test. Source data are provided in Supplementary Information.

While the dimensions of the brain extracellular space allowing movement of particles already within the parenchyma is estimated to be 38–64 nm in vivo^50^, the size of the space between overlapping astrocyte end feet allowing passage of CSF and its tracers from the perivascular spaces into the parenchyma is accepted to be approximately 20 nm^13,51^. Accordingly, we reasoned that large CSF tracers (15 nm AuNP core with a 35.91 nm average hydrodynamic diameter) could enter the perivascular space, but not subsequently efflux into the interstitial fluid (ISF) via perivascular-dependent mechanisms (Fig. 4a). This finding would therefore allow for evaluation of non-perivascular influx-dependent intraparenchymal CSF circulation patterns.

A direct histologic comparison 10 minutes after intraventricular injection of small and large CSF tracers revealed distinct particle-size dependent differences in CSF handling within the brain and spine (Fig. 4b–j). 15 nm AuNPs were primarily localized extracellularly in lateral and third ventricle periventricular regions and additional sparse extracellular 15 nm AuNP distribution was observed in cortex, midbrain, brainstem, and spinal cord regions (Fig. 4e, g–j). We did not observe cellular uptake of 15 nm AuNPs, unlike the cellular 1.9 nm AuNP uptake within the 24 functional-anatomic cell groupings. 1.9 nm AuNPs also covered significantly more total cross-sectional brain area than 15 nm AuNPs (Fig. 4f). Furthermore, 15 nm AuNPs localized to the apical surface of the lateral ventricle choroid plexus (ChP), while 1.9 nm AuNPs distributed across both the apical and basolateral surfaces 10 min post-intraventricular injection (Fig. 4k). AuNP-XRM revealed 15-nm AuNPs distributed around the optic nerve 4 h post-intraventricular injection (Fig. 4l–n), while 1.9 nm AuNPs were within the optic nerve 10 min post-injection (Fig. 4l–n).

**Fig. 5:**
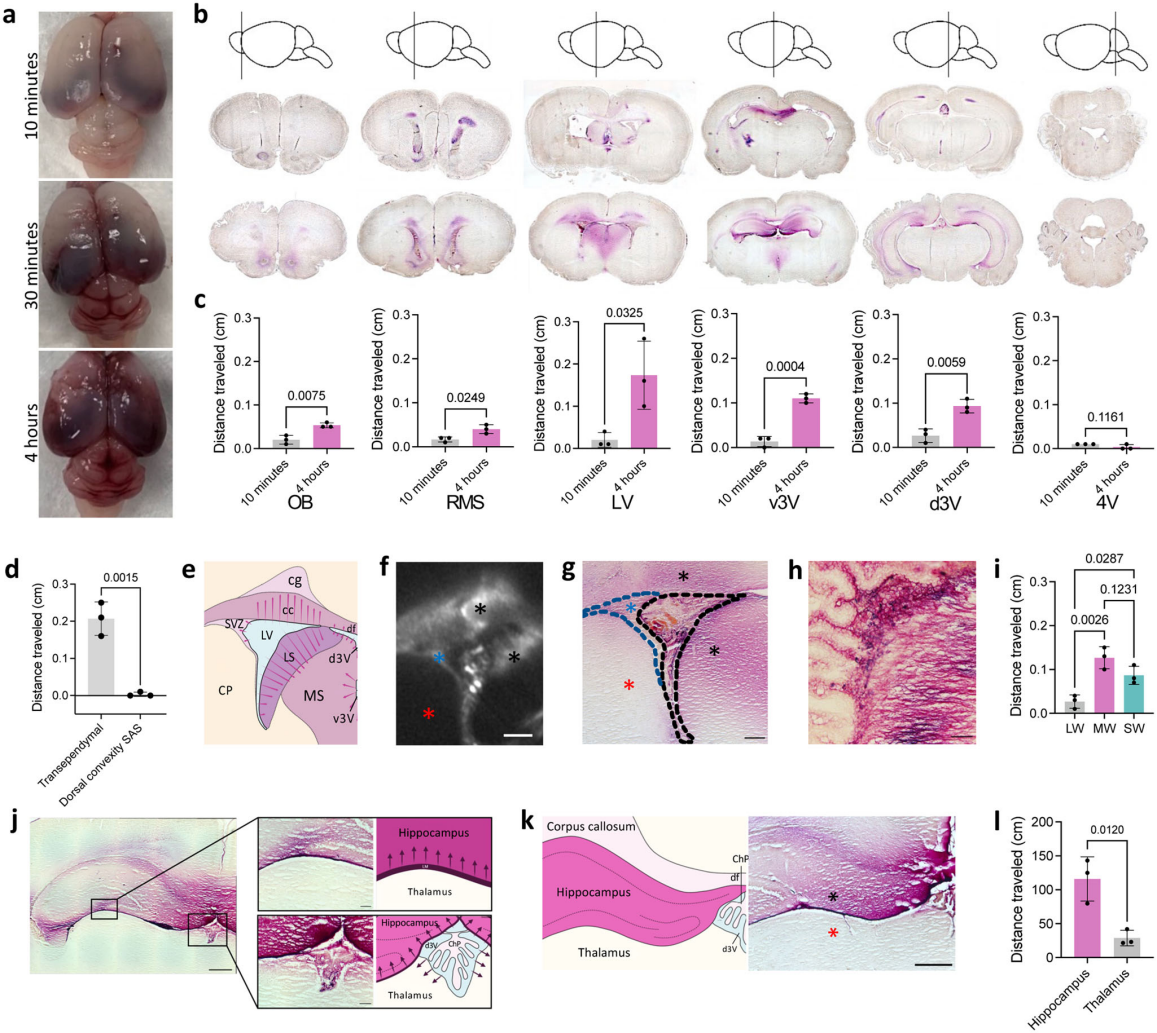
Region-specific perivascular-independent transventricular, transleptomeningeal, and transependymal circulation of large CSF tracers. **a–b** Gross (**a**) and histology (**b**) images of 15 nm gold nanoparticle (AuNP) (magenta) distribution 10 min, 30 min, and 4 h after intraventricular injection in P7 rodents. **c** 15 nm AuNP influx distance from the center of the olfactory bulb; rostral migratory stream; ependyma of the lateral ventricle medial wall (LV), ventral third ventricle (v3V), dorsal 3V (d3V); and floor of the fourth ventricle (4V) 10 minutes and 4 h post-intraventricular injection. Data are mean ± S.D., *n* = 3 rodents, unpaired two-tailed t-test. **d** Transependymal distance traveled by 15-nm AuNPs into the brain parenchyma from the LV medial wall compared to depth of penetration via influx from the dorsal convexity subarachnoid space 4 h post-intraventricular injection. Data are mean ± S.D., *n* = 3 rodents, unpaired two-tailed t-test. **e–g** Schematic (**e**), X-ray microtomography (XRM) (**f**), and histology (**g**) demonstrating transependymal 15 nm AuNP circulation through the medial (MW) and superior walls (SW) (black asterisks) and the subventricular zone (SVZ) (blue asterisk) of the right LV 4 h post-injection. There was very little 15 nm AuNP influx across the lateral wall (LW) lateral and inferior to the SVZ (red asterisk). Scalebars = 250 µm. Abbreviations: CP, caudate putamen; SVZ, subventricular zone; cg, cingulum; cc, corpus callosum; LS, lateral septal nucleus; MS, medial septal nucleus; df, dorsal fornix; LV, lateral ventricle; d3V, dorsal third ventricle; v3V, ventral third ventricle. **h** Higher magnification image of the right LV choroid plexus and MW. Scalebar = 50 µm. **i** Intraparenchymal distance traveled by 15 nm AuNPs from the LV MW, SW, and LW. Data are mean ± S.D., *n* = 3 rodents, one-way ANOVA with post hoc Tukey. **j** Histology and schematic showing 15 nm AuNP circulation into the hippocampus. scale bar = 500 µm, inset scalebar = 100 µm **k** Histology and schematic showing 15 nm AuNP influx into the hippocampus through the d3V (black asterisk) with sparse 15-nm AuNPs observed in the thalamus (red asterisk). scalebar = 1 mm. **l** 15 nm AuNP mean intensity in the hippocampus and thalamus. Data are mean± S.D., *n* = 3 rodents, unpaired two-tailed t-test **d**–**l** all observations and quantifications were made 4 h post-15 nm AuNP injection into the right LV across 3 rodents. Source data are provided in Supplementary Information. Figure 5e, j, and k were created with BioRender.com.

After identifying differences between 1.9- and 15 nm AuNP distribution patterns, we aimed to characterize large CSF tracer entrance into the parenchyma. 15 nm AuNPs were present unilaterally in the right olfactory bulb through the rostral migratory stream (RMS), SVZ, and corpus callosum 10 min post-intraventricular injection (Fig. 5a, b). There was more widespread distribution four hours post-injection (Fig. 5b, c), with 15 nm AuNPs identified bilaterally in the RMS, SVZ, corpus callosum, as well as the brain parenchyma medial to the lateral ventricles including the hippocampus more posteriorly (Fig. 5b, c). Unlike small CSF tracer distribution through cortical CSF pathways, there was little to no 15 nm AuNP influx into the parenchyma from the surface of the brain (Fig. 5d).

In contrast to the targeted functional-anatomic localization observed with 1.9 AuNPs, 15 nm AuNPs extended into the parenchyma in a more widespread, non-cellular gradient distribution from the ventricular ependyma, suggesting these large particles were transported via non-perivascular transependymal (lateral ventricle, ventral third ventricle) and transventricular (dorsal third ventricle) routes into the brain parenchyma. The transependymal movement of large CSF tracer was not uniform across the lateral ventricle walls, with preferential movement across the ependyma of the medial and superior walls of the lateral ventricles (and not the lateral wall) (Fig. 5e–i). This formed a gradient with decreasing coverage superiorly into the corpus callosum and medially into the lateral septal nucleus.

15 nm AuNPs entered the hippocampus through two routes: transventricular across the roof of the third ventricle and transleptomeningeal from the quadrigeminal cistern (Fig. 5j, k). Notably, 15 nm AuNPs originating from the leptomeninges of the perimesencephalic cisterns only extended superiorly into the hippocampus without movement inferiorly into the thalamus (Fig. 5j–l).

### Differential distribution of large CSF tracers in neonatal post-hemorrhagic hydrocephalus

In contrast to the overall decrease in 1.9 nm AuNP distribution after PHH, 15 nm AuNP distribution was increased in the olfactory bulb and corpus callosum, but there were no significant differences in the cerebellum, SVZ, or hippocampus (Supplementary Fig. 11). These differences may be due to increased transependymal CSF flow across the lateral ventricle walls^52,53^.

## Discussion

In this study, we used AuNP-XRM as a high-resolution CSF imaging method to map CSF pathways in rodents. AuNP-XRM was capable of identifying CSF pathways from the dorsal and basal aspects of the brain, the latter of which has not been extensively explored until recently due to imaging constraints^54^. This method exploits the high resolution of X-rays and high X-ray attenuation of AuNPs to identify 24 anatomically and functionally distinct cell groupings to which CSF is targeted via previously uncharacterized CSF pathways. These include basal CSF influx to cranial nerve nuclei, as well as other routes such as more widespread CSF influx to the cerebellar molecular layer and perivascular-independent transependymal, transleptomeningeal, and transventricular circulation to stem cell rich populations, including the hippocampus.

CSF circulation is not well understood. One popular paradigm is the glymphatic system, which is defined as a glial-dependent waste clearance system^13,38^. In this present study, we take an alternate view of CSF; rather than address waste clearance, we evaluate how CSF enters the brain via 1. influx from the subarachnoid space and cisterns, and 2. the ventricles to bathe specific functional-anatomic cell groupings in the brain. In addition, while CSF handling along the MCA has received intense focus in the context of glymphatics^13^, we did not observe a clear route from the MCA penetrating arteries and arterioles to the 24 functional-anatomic cell groupings, particularly those in the brainstem. Our findings have implications for areas of the brain which may require CSF delivery and interaction during development. Another important question that stems from our findings is how CSF and its solutes may interact with neurons. A recent study showed that infusion of CSF from young rodents into the brains of aged animals resulted in improved memory function^6^. This finding further highlights the importance of understanding specifically where CSF influxes into the brain, as it suggests that CSF contents, such as the fibroblast growth factor 17 (Fgf17) identified in the study, may engage in regulatory interactions with neurons and glia in the parenchyma. Other studies have shown the importance of the specific composition of CSF by developmental stage and its role in stem cell proliferation^30,31^. Taken together with our findings, these studies suggest that physiologic intraparenchymal CSF transport and influx patterns may orchestrate complex, targeted CSF delivery of key nutrients to nourish the brain as it develops and matures.

By inducing PHH, we utilize a system of known CSF circulation dysfunction to abrogate the majority of the CSF influx pathways identified in this manuscript^36,37,49^, indicating that these influx pathways are dynamic and altered in the disease state of hydrocephalus. Specifically, we observed differences in the pattern of intraparenchymal CSF distribution between control and PHH rodents, where there were decreases in AuNP delivery to 15 of the 24 identified functional-anatomic cell groupings. The finding of IVH-PHH altering intraparenchymal CSF distribution to brain regions important in learning, memory, and motor function has significant implications for CSF-mediated direct brain injury through transport of molecules/signals to neuronal and glial populations involved in these critical functions. These findings are supported by a recent study showing that congenital hydrocephalus develops as a result of impaired stem cell development^29^. Restoring the interactions between CSF and the neurons it communicates with may present a therapeutic target for hydrocephalus pathology.

In line with the co-localization of the majority of these 24 functional-anatomic cell groupings with ChAT, a key enzyme in the biosynthesis pathway of the neurotransmitter acetylcholine, the cholinergic system has also been associated with hydrocephalus in several prior studies^55,56^. In our IVH-PHH model, we identified significant differences in 1.9 nm AuNP uptake within 15 functional-anatomic cell groupings between control and PHH rodents, the majority of which are also ChAT+ regions. These findings suggest that disruption of fluid dynamics within the ventricles may affect intracerebral CSF delivery to ChAT+ cells, with further implications for cognitive dysfunction and the development of neurons within these cholinergic nuclei in hydrocephalus pathology. Previous studies have shown that cholinergic neurons express receptors for various growth factors secreted by the ChP, a subset of which are also known to promote the development of cholinergic neuronal populations both in vivo and in vitro. These growth factors specifically include cholinergic differentiation factor/leukemia inhibitory factor (CDF/LIF), insulin-like growth factors I and II (IGF-I, IGF-II), fibroblast growth factor 2 (fgf2), and ciliary neurotrophic factor (CNTF)^57–67^. We speculate that the targeted CSF pathways identified in this present study may have a role in disseminating these growth factors from their sites of secretion in the ChP of the cerebral ventricles to the microenvironment around developing cholinergic neurons. Future studies should evaluate the effect of altered ChP expression of these growth factors on cholinergic neuron differentiation and brain maturation.

We also identified CSF movement from the lateral and third ventricles into neurogenic niches, including the subventricular zone, hippocampus, and olfactory bulb (via the rostral migratory stream). The differential and regional transependymal, transleptomeningeal, and transventricular movement of large CSF tracers across ependymal walls and the leptomeninges with proximity to the hippocampus, pineal gland, hypothalamus, and corpus callosum may implicate a biological vulnerability of these structures to CSF abnormalities. The movement of CSF and CSF solutes into these areas may have a role in communication between the CSF and cells within the brain parenchyma. During development, it is possible that this transventricular movement of CSF represents a key mechanism by which nutrients and growth factors reach the hippocampus and SVZ^30,31,68–70^. This interpretation is consistent with prior studies that show growth factors, neurotransmitters, and other molecules pertinent in various signaling pathways cross the ventricle wall to interact with type A, B2, and C cells in the SVZ^30,69,70^. Changes in CSF composition and circulation dynamics in the setting of IVH-PHH may disrupt the interaction of these cells with transependymally and transventricularly transported molecules critical for development, with implications for ventricular zone disruption, neural stem cell loss, and eventual neurodevelopmental outcomes.

There are several limitations to this study. The primary limitation is the lack of specific mechanistic characterization of how neurons and other cells may interact with, take up, and metabolize CSF components, and how these processes may differ between brain regions. Prior studies have shown nanoparticle trophism is largely dependent on surface chemistry but have not implicated a preferential interaction of PEG-coated NPs with specific cell types^71,72^. Accordingly, without knowing the mechanism by which the 24 functional-anatomic cell groupings interact with 1.9 nm AuNPs, it cannot be ruled out that the CSF patterns identified in this study are affected by a trophism for the AuNPs or other tracers to these functional-anatomic cell groupings. However, we believe that this is unlikely as similar patterns of CSF tracer distribution were observed with other small molecule tracers with variable surface chemistry. Through direct review of figures in published reports of either CSF solutes or tracers administered to the CSF for other purposes, we confirm that the patterns of CSF movement are consistent with data from existing studies, although these prior studies had not specifically noted these patterns. Prior studies using intraventricular injection of radioactive transferrin in P7 rodents show similar patterns of transferrin distribution similar to the 1.9 m AuNP distribution observed here^73^. There is also evidence for 3 kDa FITC-dextran distribution along the same CSF pathways arising from the interpeduncular fossa and terminating in the brainstem nuclei identified in this study^21^. The consistency of the identified CSF pathways across an array of tracer types, CSF administration modalities, and imaging parameters suggest that the CSF pathways identified in this study are representative of biologically inherent CSF circulation patterns. Nevertheless, contextualization of the significance of these findings to our understanding CSF circulation necessitates investigation into and further appreciation of the biological mechanisms by which these patterns arise.

Additional limitations to this present study include the lack of CSF pathway characterization across a wide age range to evaluate the developmental time course for changes in CSF pathways identified between P8 and P21, the high radiation dose and long scan time of AuNP-XRM necessitating experiments to be performed ex vivo, and the lack of vascular mapping to better understand how the CSF pathways may relate to vascular hemodynamics. In addition, while we confirmed the neuronal identity of cells within a subset of the 24 functional-anatomic cell groupings and implicate a correlation with ChAT expression, further evaluation is needed to determine the specific cell subtypes that may be interacting with the CSF and whether there are trophic factors governing this interaction. Related to this point, the functional-anatomic cell groupings we identified with the small solute tracer were gray matter areas anatomically consistent with neuronal cell populations; while many cells were consistent with neurons based on morphology, we only analyzed a subset of cell groupings to confirm the neuronal cell type with cresyl violet and/or NeuN. It is possible that CSF is delivered to multiple cell types within these functional-anatomic regions. Future studies should also investigate the mechanisms and various pathophysiological aspects of IVH-PHH that may lead to the reduction of targeted CSF delivery to a subset of the identified areas of CSF-parenchyma interactions.

This work describes a CNS-wide map of functional anatomic brain and spine regions which preferentially see and handle CSF. We show the advantages of using this high-resolution global imaging modality to identify regions of interest for future CSF studies, highlighting the importance of the basal aspects of the brain to our understanding of CSF influx and circulation. These results provide a framework for the future and necessary mechanistic study of CSF-brain and CSF-spine interactions based not only on location and function, but also tracer size. This work has implications for CSF circulation in the modulation of neurogenesis during neurodevelopment, CSF circulation pathologies including hydrocephalus, and pathological basis for developmental brain pathology.

## Methods

### Animals (Rodents and zebrafish)

All experiments were approved by the Institutional Animal Care and Use Committee at Washington University in St. Louis (protocols #19-0905 and 19-1026). Sprague Dawley Rats (crl:SD 400, Charles River Laboratories, Wilmington, MA) were used in all experiments involving rats and C57BL/6J mice (WT, JAX 000664, The Jackson Laboratory, Bar Harbor, ME) were used in all experiments involving mice.

Female and male post-natal day 4–7 Sprague Dawley Rats and female and male post-natal days 8–21 C57BL/6J mice were housed with their dams in a 12 h light–dark cycle in a temperature and humidity-controlled room. Water and food were provided ad libitum for the dam.

Zebrafish were raised under standard conditions following IACUC guidelines. A casper line was crossed to the mitfa^−/−^Tg(HuC:EGFP) line and embryos were screened for EGFP signal at 24 h post fertilization (hpf). 1–5 dpf embryos were kept in 28.5C water in a 100 mm petri dish with egg water (1.5 mL stock salts (40 g Marine “Instant Ocean” Sea Salts added to 1 L distilled water) in 1 L distilled water) at a maximum density of 25 embryos/mL.

Sample size is consistent with recent studies using XRM/MicroCT for visualizing biological structures and are generally *n* = 3 per group in this study^74^. Statistical methods were not used to re-calculate or predetermine sample sizes in any experiments.

### AuNP size and zeta potential confirmation

The hydrodynamic size and morphology of 1.9- and 15 nm AuNPs (1102 and 1115, Nanoprobes, Yaphank, NY) were confirmed using transmission electron microscopy (TEM) with a JEOL JEM-1400Plus Transmission Electron Microscope (JEOL, Peabody, Massachusetts) operating at 120 kV. A 2 μL sample was placed on an ultrathin lacey carbon grid (Formvar/Carbon 200 mesh, Copper, Ted Pella Inc., Redding, California) and allowed to sit for 5 min before removal of the droplet via wicking with a Chemwipe and vacuum drying. Hydrodynamic diameter and zeta potential of the suspensions were determined with the Malvern Zetasizer Nano-ZS ZEN 3600 (Malvern, Malvern, United Kingdom) at 25 °C. Particle measurements were performed in a 1 cm path-length quartz cuvette and a folded capillary zeta cell (Malvern Instruments Ltd, Malvern, United Kingdom), respectively. Samples were highly diluted (*c* < 0.1 wt%) to prevent multiple scattering. A triplicate of the sample was diluted in Dulbecco’s phosphate-buffered saline (DPBS) for dynamic light scattering and deionized water for zeta potential measurements. Z-average size and polydispersity index of the AuNPs were obtained with an average of 12 runs. TEM was performed to validate morphology and size of the AuNPs.

### Intraventricular hemorrhage induction

The workflow for these experiments is outlined in Fig. 1a, b. In IVH-PHH experiments, rats from the same cage were randomly assigned to receive different treatments (aCSF control vs. Hb injection). All rodents receiving P7 intraventricular 1.9-nm AuNP injections and a subset of those receiving P7 15 nm AuNP injections first received either aCSF or Hb pre-injection at P4 to allow for direct comparison of CSF circulation patterns to IVH-PHH conditions^36,37,49^. The purpose of the Hb injection was to induce IVH-PHH; the purpose of the aCSF injection was to serve as a volume control for the Hb injection. No changes in the size of the ventricular system were observed in aCSF-injected rodents 72 h post-injection, however all Hb-injected rodents developed ventriculomegaly.

To inject aCSF and Hb, anesthetized (isoflurane 2.5–3% induction and 1.5% maintenance) P4 rats were fixed in a stereotaxic frame (Model 900LS Lazy Susan, KOPF Instruments, Tujunga, CA). A 2.5 mm midline incision was made and a 0.3cc syringe with a 30-gauge needle was inserted into the right lateral ventricle (1.5 mm lateral, 0.4 mm anterior, and 2.0 mm deep). A small volume (20 μL) of artificial CSF (aCSF) (Tocris Bioscience, Bristol, UK) or hemoglobin (150 mg/ml diluted in aCSF) (EMD Millipore Corp, Burlington, Massachusetts) was injected at a rate of 8000 nL/min using a micro-infusion pump (World Precision Instruments, Sarasota, FL) to create the aCSF control and IVH-PHH conditions respectively^36,49^. The needle was left in for 5 minutes post injection to prevent backflow. The incision was closed with 6-0 Ethilon suture (Ethicon Inc, Raritan, NJ). Rodents recovered from anesthesia and were returned to their cage with the mother.

### MRI quantification of ventricle volume

MRI was obtained 72 h after intraventricular aCSF or Hb injection and images were acquired using a 4.7 T Varian MRI scanner (Varian Inc., Palo Alto, California) with T2-weighted fast spin echo sequences (repetition time 3000/echo time 27.50 ms, 3 averages, field of view 18.0 mm × 18.0 mm, matrix 128 × 128, 24 axial slices, and 0.50 mm thick). Rodents were anesthetized using isoflurane (2.5–3% induction, 1.5% maintenance), placed in a plastic holder and kept warm using a heated air blower. Lateral ventricles were segmented and quantified using ITK-SNAP (Version 4.0.0 beta). 

### Intraventricular AuNP injection

1.9 or 15 nm AuNPs (1102 and 1115, Nanoprobes, Yaphank, NY) were constituted in aCSF at a concentration of 200 mg/mL for intraventricular injection. Anesthetized P7 rats underwent intraventricular injection of 20 µl of 1.9 or 15 nm AuNPs as per protocol above with the following coordinates from bregma (1.7 mm lateral, 0.5 mm anterior, and 2.0 mm deep)^37^. 10 min post-injection, rodents were perfusion-fixed with 10 mL of ice-cold PBS followed by 10 mL of 4% paraformaldehyde (PFA) at 4 °C.

### XRM acquisition and analysis

Following perfusion, the entire rat was placed in 4% PFA at 4 °C overnight, transported to the imaging facility, embedded in 2% agarose, and imaged within 24–72 h of sacrifice with a Zeiss Versa 520 X-ray microscope (Carl Zeiss Imaging, White Plains, NY) using a 0.4× flat panel detector. X-ray source was tuned to 50 KV at 4 W to optimally excite AuNPs. For samples requiring larger fields of view, we used the Versa’s wide or vertical stitch functions, as needed. A total of 1601 projections were acquired, reconstructed, and tomograms were visualized in either Zeiss XM3DViewer 1.2.8 (Carl Zeiss Imaging, White Plains, NY) or in Dragonfly 2020.1 (Object Research Systems, Montreal, Quebec).

Processed images from 1.9 nm AuNP-XRM were systematically reviewed and quantified; for standardization, images were numbered by their location relative to bregma, so the location of the selected images was consistent across all three rodents. Three areas were randomly selected in each anatomic location, and the mean gray intensity of these areas was calculated using FIJI/ImageJ (Version 2.3.0/1.53q) and averaged to obtain one mean gray intensity measurement per area per rodent. The mean gray intensity of the agarose background was calculated for each image and subtracted out from the mean gray intensity of the anatomic location to standardize the images. Mean intensities for the adjacent region obtained for comparison in Supplementary Fig. 1 were quantified following a similar procedure. Specifically, an area 1–5 µm from the edge of each anatomic area was selected and the mean gray intensity measured. The mean gray intensity of the agarose background was also subtracted out from this value for standardization.

### Skull stripping and semi-automatic segmentation

The XRM scan from one rodent was registered to the Duke Wistar rat atlas in-house with 4dfp tools (http://4dfp.readthedocs.io). Images were downsampled to 40 μm and transformation was computed to register images to the P8 average gradient echo image of the Duke Wistar atlas^42^. This transformation was then applied to the full-resolution images. The co-registered atlas image (included only brain) was then used as a mask such that only the intra-cranial contents of each image remained. After skull-stripping, regions of intraparenchymal CSF were semi-automatically segmented to reveal the same patterns of AuNP distribution as those identified manually (Fig. 1c, e, f insets).

### AuNP localization using light microscopy

After XRM image acquisition, the rodents were removed from the agarose and left in 4% PFA overnight. The brains and spines were harvested and washed 2× in PBS for 1 h each, then immersed in 30 and 50% ethanol for 30 min each, before being transferred to 70% ethanol for 24 to 72 h at 4 °C prior to xylene and paraffin processing. The brains and spines were embedded in paraffin, and 10 μm thick slices were sectioned in either the coronal or sagittal planes using a microtome to examine the distribution of AuNPs. Following overnight incubation at 60 °C, sections were soaked in xylene for 20 min and mounted with Permount mounting medium (#SP15-100, Thermo Fisher Scientific, Waltham, MA) for light microscopy localization of AuNPs.

For the histological quantification of the data presented in Fig. 4f, sections from the anterior lateral ventricles were obtained across 3 rodents each for both 1.9 and 15 nm AuNP-injected rodents. The total brain area with AuNP penetration was outlined and quantified using FIJI. For the quantification of 15 nm AuNP mean intensity performed in Fig. 5l, photomicrographs were opened in FIJI and converted to greyscale before being inverted. The mean intensity of three random regions each of the hippocampus and thalamus was obtained for three separate rodents and averaged within each rodent to obtain one measurement from the hippocampus and another measurement from the thalamus for each rodent. For quantification of the data presented in Fig. 5c, the straight-line distance from the center of the olfactory bulb or rostral migratory stream, and/or the straight-line distance from the ependyma of the medial wall of the lateral ventricle, ventral third ventricle, dorsal third ventricle, or fourth ventricle to the most distal edge of the continuous 15 nm AuNP gradient was measured across three separate rodents. For quantification of the data presented in Fig. 5d, the straight-line distance from the ependyma of the medial wall of the lateral ventricle to the most distal edge of the continuous 15 nm AuNP gradient was measured for transependymal/transventricular influx. The straight-line distance from the dorsal surface of the brain at the anterior-posterior level of the anterior lateral ventricle to the most distal edge of the continuous 15 nm AuNP gradient into the parenchyma was measured for dorsal convexity SAS influx. Quantifications were performed across three separate rodents. For quantification of the data presented in Fig. 5i, the straight-line distance from the ependyma of the medial wall, superior wall, and lateral wall of the lateral ventricle to the most distal edge of the continuous 15 nm AuNP gradient was measured across three rodents.

To co-stain gold sections with cresyl violet, sections were hydrated through descending grades of alcohol (100, 95, 70, 50, and 30%) to double distilled water (DDW) following xylene and immersed in FD Cresyl Violet Solution (#PS102-02, FD NeuroTechnologies, Columbia, MD) for 5 min. Sections were left in running DDW for 5 min then differentiated and rehydrated through ascending grades of alcohol (30, 50, 70, 95, and 100%). Sections were then cleared in xylene for 20 min before mounting with Permount mounting medium (#SP15-100, Thermo Fisher Scientific, Waltham, MA).

### Anatomical analyses

Anatomical analyses identifying the location of AuNP circulation were performed using Brain Maps 4.0^40^ and The Rat Brain in Stereotaxic Coordinates (Seventh Edition)^41^.

### ICM 1.9 nm AuNP injection

1.9 nm AuNPs (1102, Nanoprobes, Yaphank, NY) were constituted in aCSF at a concentration of 400 mg/mL. Anesthetized P7 rats underwent ICM injection of 5 µl AuNP solution over 10 min (0.5 µL/min). Briefly, a 5 mm midline incision was made in the skin over the cisterna magna and the underlying muscle was gently separated to expose the cisterna magna. A 0.3cc syringe with a 30-gauge needle was tilted at a 30–45° angle and inserted into the cisterna magna. AuNPs were injected using a micro-infusion pump (World Precision Instruments, Sarasota, FL). The needle was left in for 5 min post injection to prevent backflow. Rats were sacrificed 10 min after 1.9 nm AuNP injections and intracardially perfused with 10 mL of ice-cold PBS followed by 10 mL of 4% PFA. Brains were harvested and left in 4% PFA overnight before processing and embedding for histological analysis.

### Red-Dextran/TMR ICM injection

3 kDa MW Red-Dextran/Tetramethylrhodamine (D3307, ThermoFisher Scientific, Waltham, MA) were constituted in aCSF at a 0.25% concentration. Anesthetized native P7 rats underwent ICM injection of 5 µl Red-Dextran TMR over 10 min (0.5 µL/min) following the procedure above. Rats were sacrificed 10 min after the end of the injection and either intracardially perfused with 10 mL of ice-cold PBS followed by 10 mL of 4% PFA or sacrificed with cervical dislocation and dropped fixed. Brains were harvested and left in 4% PFA overnight before processing and embedding for histological analysis. Following sectioning, DAPI counterstaining was performed prior to fluorescent microscopy analysis. Sections were soaked in xylene to remove paraffin, before hydration through descending grades of alcohol (100, 95, 70, 50, and 30%) to DDW. Incubation with DAPI reagent in a 1:500 dilution was performed. After 7 min in DAPI, sections were washed in PBS 5 times for 5 min and mounted with ProLong Gold Antifade Mountant (#P36930, Thermo Fisher Scientific, Waltham, MA).

### CellTracker injections

CellTracker Green CMFDA Dye (C7025, ThermoFisher Scientific, Waltham, MA) was constituted in aCSF at a 0.1% concentration. Anesthetized native P8 mice underwent ICM injection of 2 µl CellTracker over 10 min (0.2 µL/min), while P21 mice underwent injection of 5 µL over 10 min (0.5 µL/min). Mice were sacrificed 10 min after the end of the injection and intracardially perfused with 10 mL of ice-cold PBS followed by 10 mL of 4% PFA. Brains were harvested and left in 4% PFA overnight before processing and embedding for histological analysis. Following sectioning, DAPI counterstaining was performed prior to fluorescent microscopy analysis. Sections were soaked in xylene to remove paraffin, before hydration through descending grades of alcohol (100, 95, 70, 50, and 30%) to DDW. Incubation with DAPI reagent in a 1:500 dilution was performed. After 7 min in DAPI, sections were washed in PBS 5 times for 5 min and mounted with ProLong Gold Antifade Mountant (#P36930, Thermo Fisher Scientific, Waltham, MA). One additional P8 mouse brain underwent iDISCO+ brain clearing 10 min after ICM CellTracker injection^75^. Briefly, 10 min post-CellTracker injection, the mouse was sacrificed via intracardial perfusion with 5 µL Lycopersicon esculentum (Tomato) Lectin (LEL, TL), Texas Red (L32482, ThermoFisher Scientific, Waltham, MA) followed by 10 mL of ice-cold PBS followed by 10 mL of 4% PFA. The brain was removed from the skull and placed in 4% PFA overnight before it was dehydrated in steps of 20, 40, 60, 80, and 100% methanol 2× for 1 h each. Putative delipidation was done using 2×15 min incubations in dichloromethane. Finally, samples were RI matched in dibenzyl ether and imaged using a Zeiss Lightsheet 7 (Carl Zeiss Imaging, White Plains, NY) using a 5×/0.16 air objective and 5×/0.1 illuminators.

### Zebrafish injections

Glass capillaries (TW100F-4, World Precision Instruments, Sarasota, FL) were pulled to desired shape using a Sutter Inst. P-97 model Micropipette Puller (Sutter Instrument, Novato, CA). At 96 hpf, the fish were anesthetized in accordance with IACUC guidelines with tricaine methanesulfonate. During anesthetization, the tip of the pulled capillary was broken, and the capillary was loaded with an 8 mg/mL Rhodamine B isothiocyanate–Dextran 70 kDa (R9379, Millipore Sigma, Burlington, MA) in 1× aCSF. The microneedled capillary was loaded into a micromanipulator and the larval fish were placed on an agarose injection plate (under design secrecy) and oriented dorsal side up. The needle targeted the diencephalic ventricle, using the dorsal point along the midsagittal line along the border of the tectum and the cerebellum as a landmark. 1 nL of the rhodamine solution was injected into the ventricles and the fish were returned to regular egg water. After 90 min, the fish were imaged using the VAST Bioimager (Union Biometrica, Holliston, MA) and a fluorescent confocal microscope (Carl Zeiss Imaging, White Plains, NY). aCSF used in zebrafish experiments contained: 125.5 mM NaCl, 20 mM NaHCO3, 2.4 mM KCl, 0.5 mM KH2PO4, 1.1 mM CaCl2•2H20, 0.85 mM MgCl2•6H20, 0.5 Na2SO4, 5 mM glucose; pH 7.4.

### In vivo Gd-DOTA-enhanced MRI

P7 rodents were anesthetized with isoflurane (2.5–3% induction, 1.5% maintenance) and 0.5 µl of gadoterate meglumine solution (5 mmol/mL Dotarem, Guerbet, Princeton, NJ) diluted in aCSF was injected into the right lateral ventricle using stereotaxic coordinates from the bregma (1.7 mm lateral, 0.5 mm anterior, 2.0 mm deep). Rats were then placed in the Bruker 9.4T MRI (Bruker, Billerica, MA) and imaged 30 min post injection using a Bruker CryoProbe 4-channel array coil (Bruker, Billerica, MA) and the following T1-weighted parameters: repetition time 800/echo time 7.8195 ms, 4 averages, field of view 15.0 × 15.0 mm, matrix 256 × 256, 22 axial slices, 1.00 mm thick, and 14 sagittal slices, 1.0 mm thick. Images were analyzed using ITK-Snap. Regions with very high Gd-DOTA concentrations can appear dark on T1-weighted images because the image contrast becomes dominated by negative contrast from unavoidable T2* effects, while regions with lower concentrations are dominated by positive contrast from T1 effects and thus appear bright. Areas of the brain without tracer penetration are unchanged and appear gray.

### Immunohistochemistry

NeuN and ChAT co-localization experiments (Supplementary Figs. 5 and 9) were performed on rodents with ICM Red Dextran/TMR injection to allow for co-localization. All immunohistochemistry (IHC) in Supplementary Figs. 5 and 9 was performed on rodents with no AuNP injection as AuNP’s block light precluding chromogen or fluorophore-based imaging.

For IHC experiments in Supplementary Figs. 5 and 9, P7 rodents were sacrificed 10 min after ICM Red Dextran/TMR injection with intracardiac perfusion with 10 mL ice-cold PBS followed by 10 mL of 4% PFA at 4 °C. For IHC experiments in Supplementary Figs. 2 and 8, native P7 rodents were anesthetized using 3% isoflurane and perfused with 10 mL ice-cold PBS followed by 10 mL 4% PFA at 4 °C. Brains were harvested and left in 4% PFA overnight before being washed 2× in PBS for 1 h, then immersed in 30 and 50% ethanol for 30 min each, before being left in 70% ethanol for 72 h at 4 °C followed by xylene and paraffin processing. The brains were embedded in paraffin, and 10 μm thick slices were sectioned in either the coronal and sagittal planes and left in the incubator at 60° overnight. Sections were then soaked in xylene to remove paraffin, before hydration through descending grades of alcohol (100, 95, 70, 50, and 30%) to DDW. Antigen retrieval was performed using a pH 6.0 citrate buffer (Sigma-Aldrich, St. Louis, MO) by microwaving in a 1:1000 solution in DDW for 30 min. Slides were cooled to room temperature, rinsed with PBS and blocked in 5% normal goat serum, 2.5% BSA, 0.5% TX-100 in PBS for one hour. Sections were incubated with appropriate dilutions of primary antibodies (1:200 dilution of anti-ChAT (#178850, Abcam, Cambridge, MA), 1:200 dilution of anti-NeuN (#177487, Abcam, Cambridge, MA), 1:200 dilution of anti-S100β (#52642, Abcam, Cambridge, MA), or 1:200 dilution of anti-GFAP-Cy3 (#C9205, Sigma-Aldrich, St. Louis, MO) in PBS with 1% BSA and 0.5% TX-100 (TBST) overnight at 4 °C. Sections were then rinsed in PBS 6 times for 5 min each followed by incubation for 90 min at room temperature with goat anti-rabbit IgG Alexa Fluor 594 (#A32740, ThermoFisher Scientific, Waltham, MA) or goat anti-mouse IgG Alexa Fluor 488 (#A11008, ThermoFisher Scientific, Waltham, MA) secondary antibody diluted in 1:2000 TBST. Sections were then rinsed in PBS 5–6 times for 5 min each at room temperature followed by incubation with DAPI reagent in a 1:500 dilution. After 5 min in DAPI, sections were washed in PBS 3 times for 5 min and mounted with ProLong Gold Antifade Mountant (#P36930, Thermo Fisher Scientific, Waltham, MA). Histological evaluation was performed single blinded, by a trained observer without knowledge of the treatment.

For quantification of IHC performed in Supplementary Fig. 5, confocal photomicrographs were taken from threee areas of each functional-anatomic cell grouping evaluated. In Supplementary Fig. 5, the 1. number of NeuN+ cells that were also RD/TMR+ and 2. Number of RD/TMR+ cells that were also NeuN+ were counted and averaged across the three areas to obtain one measurement per functional-anatomic cell grouping. This was repeated to obtain measurements across three total rodents.

### Statistical analysis

Rodents were randomly assigned to receive aCSF or Hb injection to create the control and PHH groups. Statistical methods were not used to recalculate or predetermine sample sizes. Experimenters were blinded to the identity of experimental groups where possible and/or relevant. Associations between two continuous variables were assessed using an unpaired t-test, and associations between more than two continuous variables were assessed using a one-way ANOVA with post hoc Tukey. Mean intensity for each anatomic location was compared to the other anatomic locations, across three different rodents. All tests were two-tailed, and *p*-values of less than 0.05 were considered statistically significant. All analyses were performed using Microsoft Office Excel (Version 16.36) or GraphPad Prism (Version 9.0.0).

### Reporting summary

Further information on research design is available in the Nature Portfolio Reporting Summary linked to this article.

## Supplementary information

Supplementary Information

Peer Review File

Reporting Summary

## Data Availability

The datasets generated and/or analyzed during the current study are available in the online version of this paper. XRM, MRI, and other raw data files are available from the corresponding author on reasonable request and will be provided within a reasonable timeframe. The “mapZebrain” atlas used for zebrafish anatomic analyses is available at: https://mapzebrain.org/atlas/2d. In-house 4dfp tools used to perform registration for skull stripping are available here: http://4dfp.readthedocs.io Source data are provided with this paper.

## Source data

Source Data

## Code availability

The code used to register a representative image from this study to an atlas image (10.1016/j.neuroimage.2012.05.041) is available at the following link: https://github.com/hackercd/Pan_Registration. A combination of Matlab, Python, and in-house tools (“4dfp” image processing tools, https://4dfp.readthedocs.io/en/latest/) was used.
