# Supplementary material for: Gold nanoparticle-enhanced X-ray microtomography of the rodent reveals region-specific cerebrospinal fluid circulation in the brain
Source: Nat Commun. 2023 Jan 27;14:453. doi: 10.1038/s41467-023-36083-1 (PMC9883388; doi:10.1038/s41467-023-36083-1)
Supplement: Supplementary file 1 — Supplementary Information [file 41467_2023_36083_MOESM1_ESM.pdf]

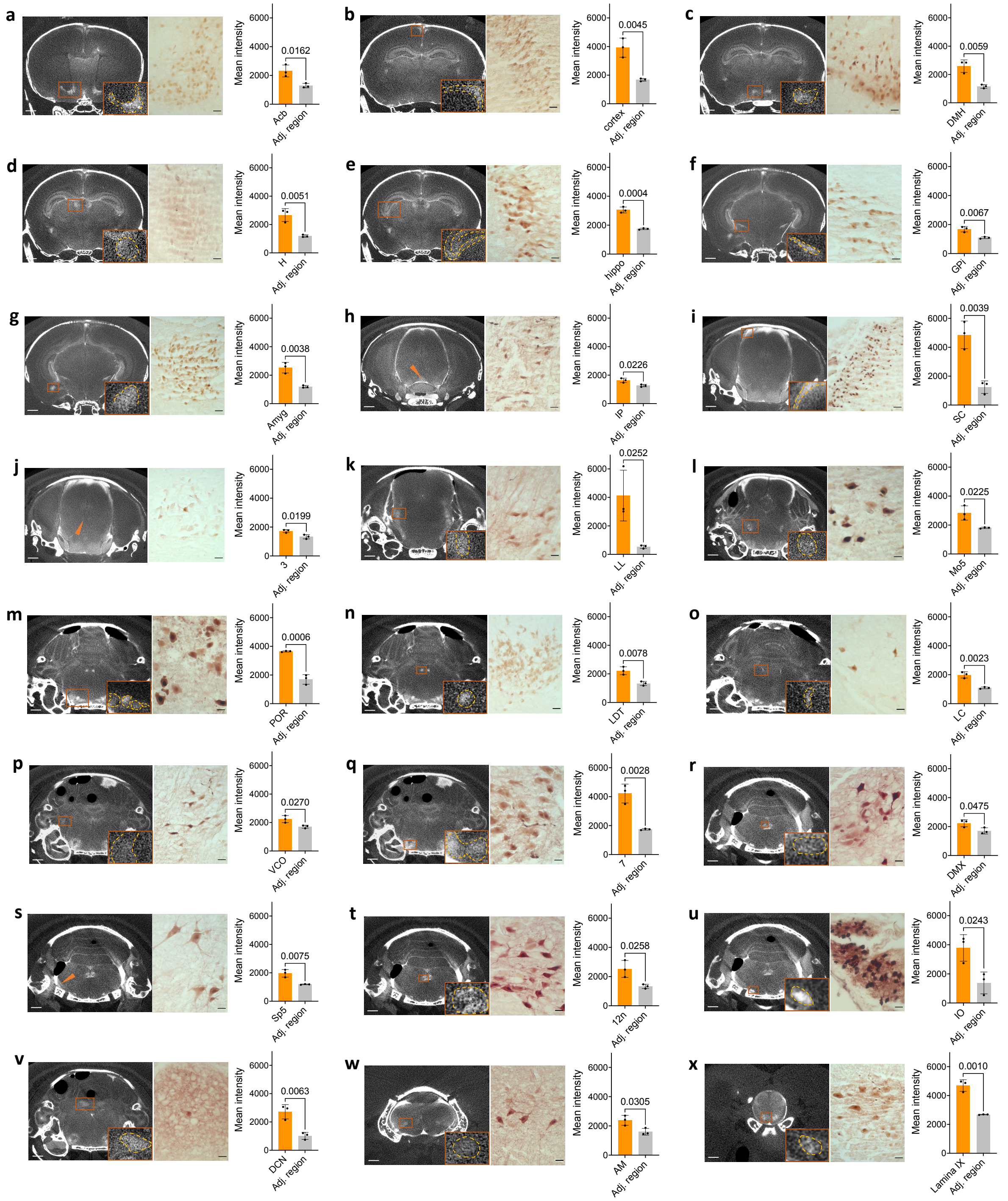

Supplementary Figure 1. Distribution of intraventricularly injected small CSF tracers to functional-anatomic cell groupings in the brain and spinal cord . a-w, X-ray microtomography (XRM) images, histology, and quantification of the mean intensity of 1.9-nm gold nanoparticles (AuNPs) showing 1.9-nm AuNP distribution in the nucleus accumbens (acb) (a), neocortex (b), dorsal medial hypothalamus (DMH) (c), habenula (H) (d), hippocampus (hippo) (e), globus pallidus internus (GPi) (f), amygdala (Amyg) (g), interpeduncular nucleus (IP) (h), superior colliculus (SC) (i), oculomotor nucleus (3) (j), lateral lemniscus (LL) (k), motor nucleus of the trigeminal nerve (Mo5) (l), periolivary region (POR) (m), laterodorsal tegmentum (LDT) (n), locus coeruleus (LC) (o), ventral cochlear nucleus (VCO) (p), facial nerve nucleus (7) (q), dorsal motor nucleus of the vagus nerve (DMX) (r), spinal trigeminal nucleus (Sp5) (s), hypoglossal nucleus (12n) (t), inferior olivary nucleus (IO) (u), deep cerebellar nuclei (DCN) (v), nucleus ambiguus (AM) (w), and lamina IX of the spinal cord (IX) (x). All 24 regions were significantly enhanced on the XRM images compared to surrounding parenchyma and had visible 1.9-nm AuNP interaction (brown) with cells populations on histology. Data are mean ± S.D., n = 3 rodents, unpaired two-tailed t-test. XRM scalebar = 1 mm, histology scalebars = 25 μm. Supplementary figure 1t is the same subfigure provided in Main Figure 2e.

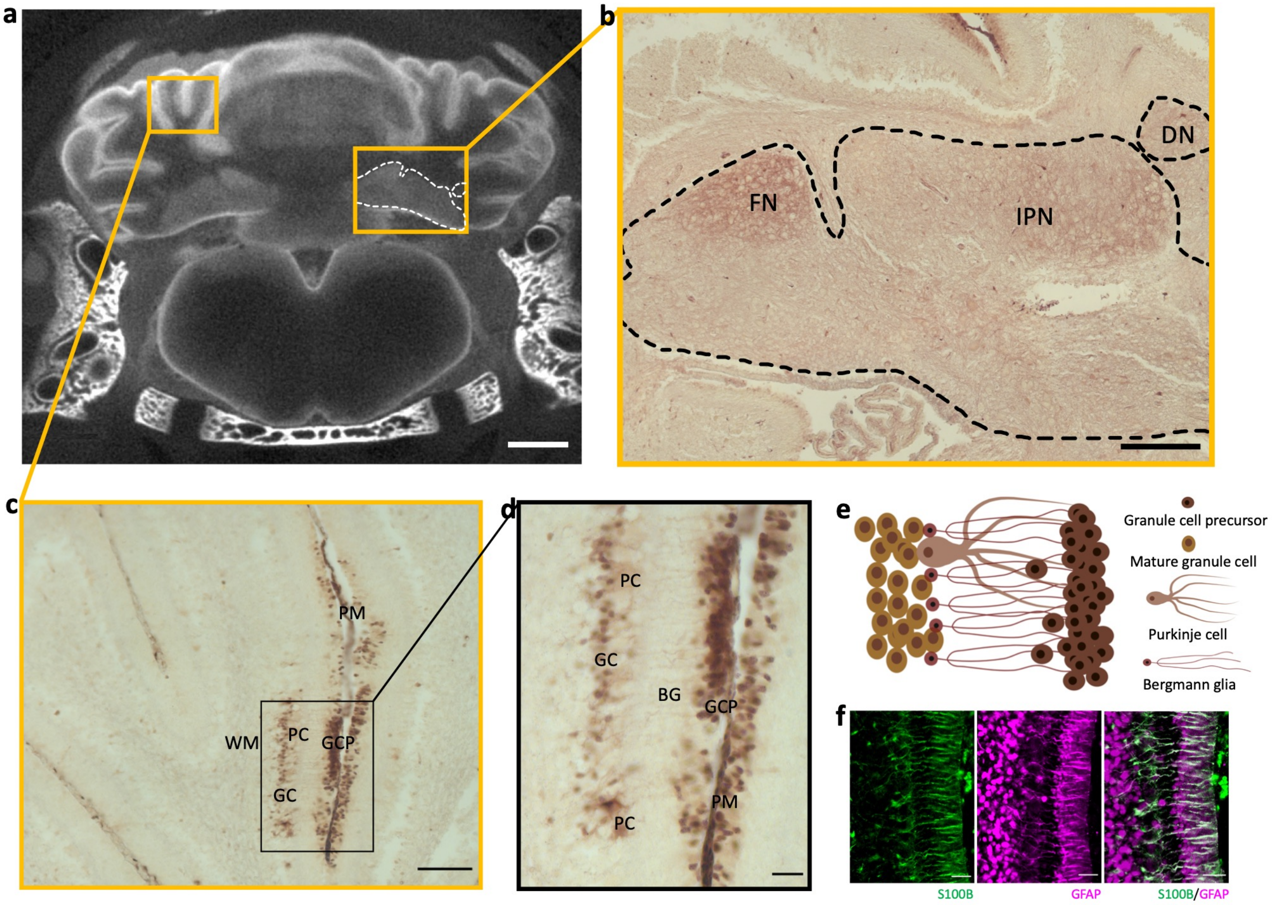

Supplementary Figure 2. Intraventricularly injected CSF tracers circulate in and around the cerebellum. **a,** Gold nanoparticle enhanced X-ray microtomography (AuNP-XRM) showing distribution of 1.9-nm AuNPs in the cerebellum 10 minutes after injection into the right lateral ventricle of P7 aCSF control rodents. 1.9-nm AuNP distribution in the deep cerebellar nuclei is indicated with a white dashed line. scalebar = 1 mm. **b,** Representative histology showing 1.9-nm AuNP distribution in deep cerebellar nuclei (black dashed line), including the fastigial nucleus (FN), interposed nucleus (IPN), and dentate nucleus (DN). scalebar = 200 μm. **c-d,** Representative histology showing 1.9-nm AuNP distribution in the leptomeningeal invaginations between the cerebellar folia (PM, pia mater), granule cell precursors (GCP) and granule cell layer (GC). 1.9-nm AuNPs were also observed in Purkinje cells (PC) and fibers of Bergman glia (BG). C scalebar = 100 μm, d scalebar = 25 μm. **e,** Schematic showing organization of cerebellar cells observed interacting with 1.9-nm AuNPs in c and d. **f,** S100 calcium binding protein B (S100B) (green) and glial fibrillary acidic protein (GFAP) (magenta) immunohistochemistry showing organization of BG and other glia in the cerebellum. scalebars = 50 μm. All data are representative from 3 rodents.

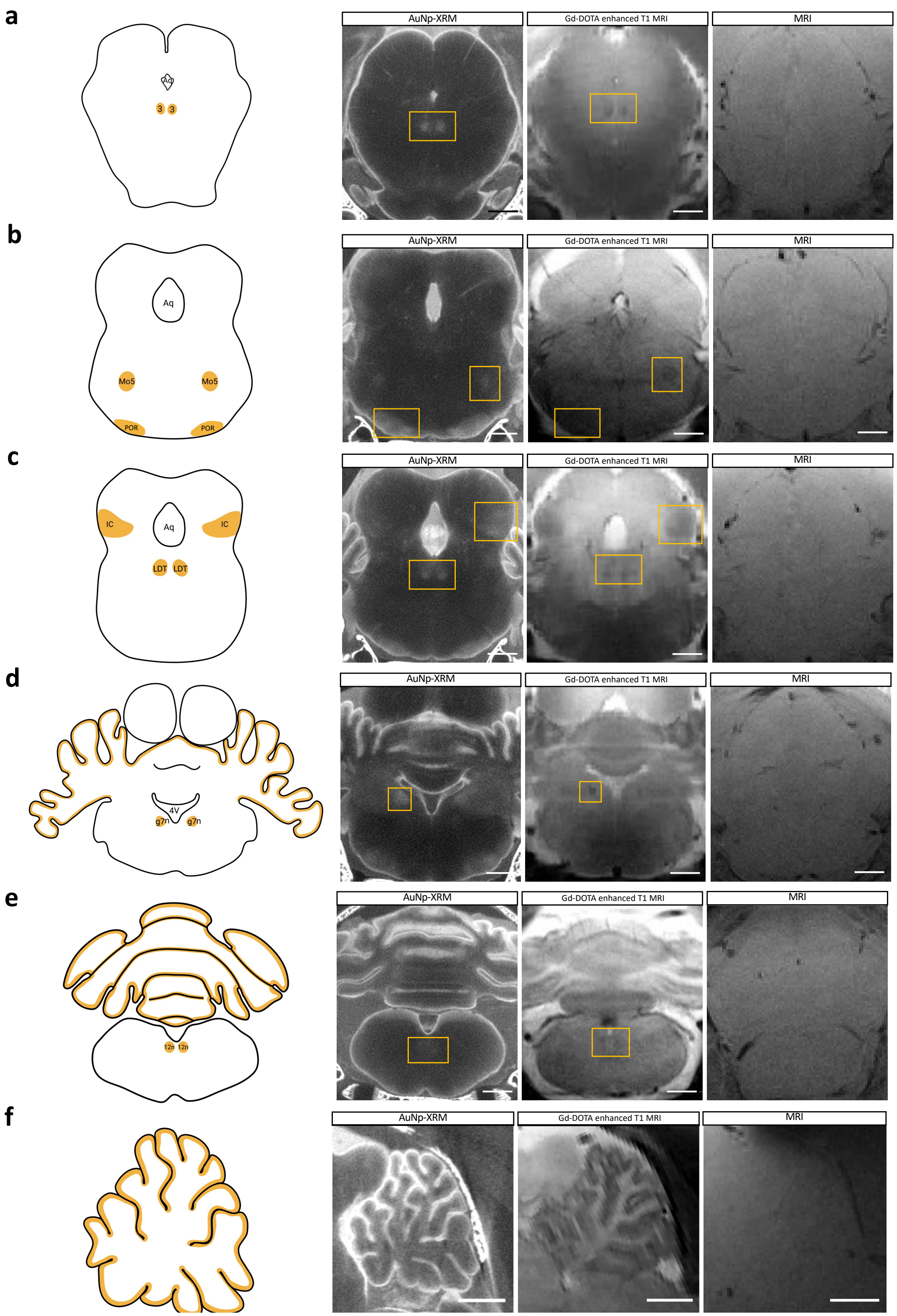

Supplementary Figure 3. Gold nanoparticle-enhanced X-ray microtomography (AuNP-XRM) demonstrates similar patterns of intraparenchymal CSF tracer distribution when compared to *in-vivo* Gd-DOTA-enhanced T1 MRIs. a-f, AuNP-XRM (middle left) performed 10 minutes after 1.9-nm AuNPs were injected into the right lateral ventricle of P7 aCSF control rodents and *in-vivo* Gd-DOTA-enhanced T1-weighted MRI (middle right) performed 30 minutes after Gd-DOTA injection into the right lateral ventricle of P7 rodents show similar patterns of tracer distribution in the oculomotor nucleus (3) (a), motor nucleus of the trigeminal nerve (Mo5) (b), periolivary region (POR) (b), inferior colliculus (IC) (c), laterodorsal tegmentum (LDT) (c), genu of the facial nerve (g7n) (d), cerebellum (d, e, f), and hypoglossal nucleus (12n) (e). Schematics showing locations of cell groupings and nuclei with tracer distribution are provided for reference (far left). A T1-weighted MRI performed without Gd-DOTA contrast agent is shown for comparison (far right). scalebars = 1 mm. All data are representative of 3 rodents.

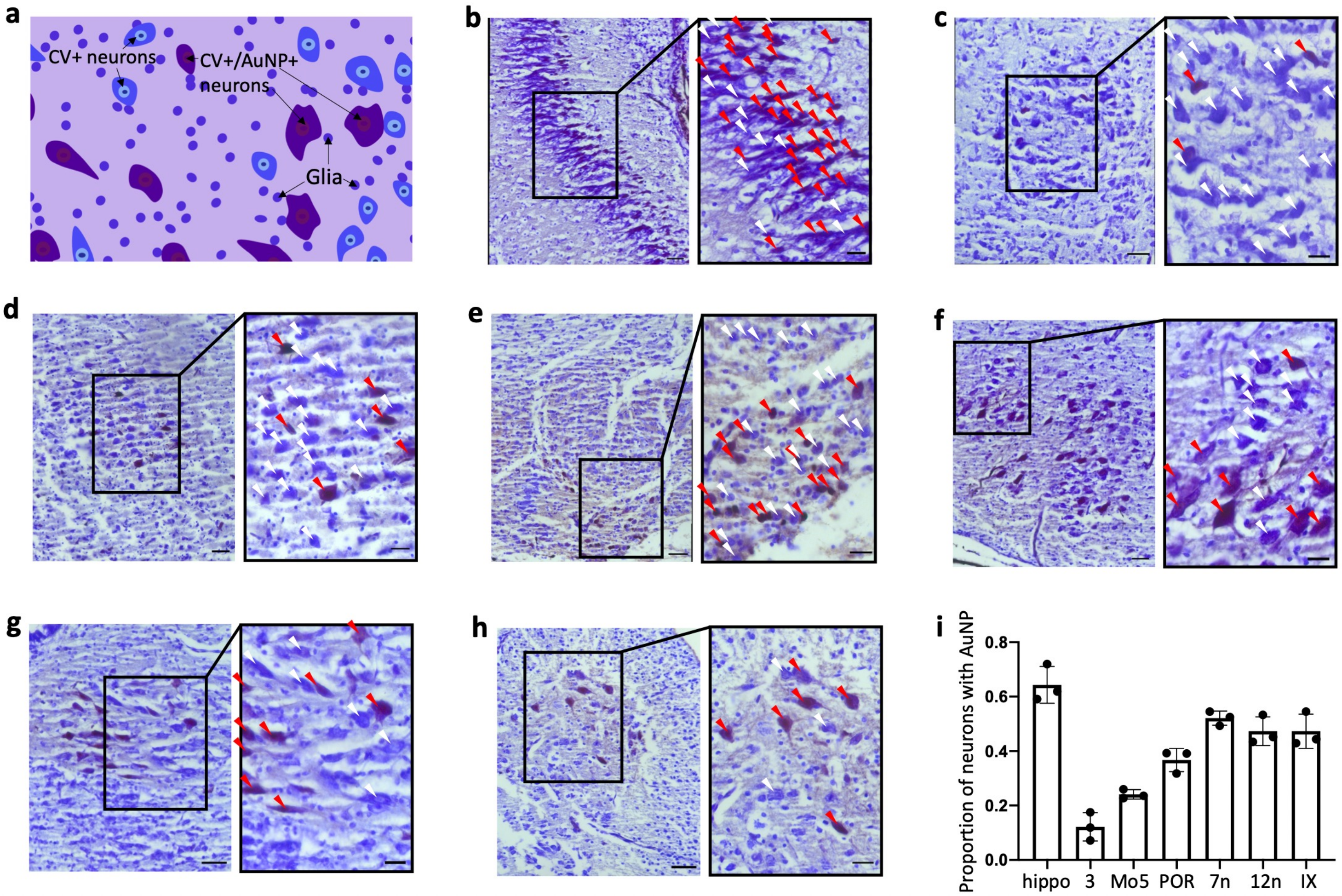

Supplementary Figure 4. 1.9-nm gold nanoparticle (AuNP) interactions with cells in brain and spinal cord nuclei is not uniform. a, Representative schematic showing distribution of neurons that are stained with cresyl violet (CV) only (CV+), CV and 1.9-nm AuNPs (AuNP+/CV+), and CV-stained glia. CV+ neurons are represented in blue while CV+/AuNP+ neurons are violet. Differentiation between neurons (large cell body) and glia (punctate) was performed based on morphology. **b-h,** Representative photomicrographs showing CV co-staining with 1.9-nm AuNPs on a section of the hippocampus (b), oculomotor nucleus (c), motor nucleus of the trigeminal nerve (d), periolivary region (e), facial nerve nucleus (f), hypoglossal nucleus (g), and lamina IX of the spinal cord (h) 10 minutes after 1.9-nm AuNP injection into the right lateral ventricle of P7 aCSF control rodents. Neurons stained with CV only are labeled with white arrowheads while neurons stained with CV and 1.9-nm AuNPs are labeled with red arrowheads. i, 1.9-nm AuNPs were observed in 64% of the CV+ neurons in the hippocampus (hippo), 12% of the CV+ neurons in the oculomotor nucleus (3), 25% of the CV+ neurons in the motor nucleus of the trigeminal nerve (Mo5), 37% of the CV+ neurons in the periolivary region (POR), 52% of the CV+ neurons in the facial nucleus (7n), 47% of the CV+ neurons in the hypoglossal nucleus (12n), and 47% of the CV+ neurons in lamina IX of the spinal cord (IX). Data are mean ± S.D., n = 3 rodents. scalebars = 50 μm, inset scalebars = 25 μm.

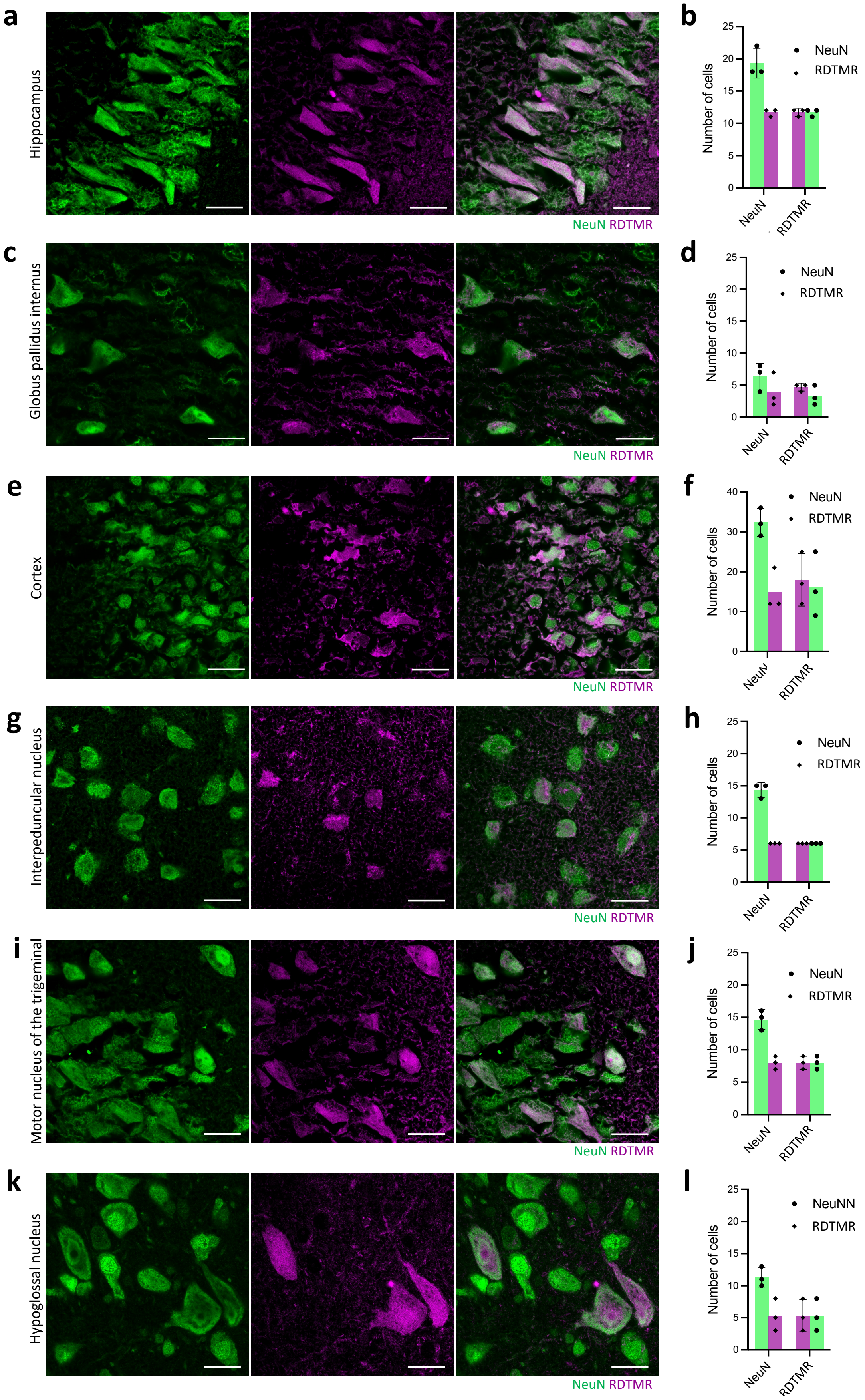

Supplementary Figure 5. Cells that take up 3 kDa Red-Dextran/Tetramethylrhodamine (RDTMR) injected into the cisterna magna are predominantly neuronal. NeuN (green) co-localization with RDTMR (magenta) in the hippocampus (a), globus pallidus internus (c), cortex (e), interpeduncular nucleus (g), motor nucleus of the trigeminal nerve (i), and hypoglossal nucleus (k). Scalebars = 25 μm. Quantification of 1. the total number of NeuN+ cells (far left green bar) and the number of NeuN+ cells that are also RDTMR+ (middle left magenta bar), and 2. the total number of RDTMR+ cells (middle right magenta bar) that are also NeuN+ (far right green bar). Quantifications were performed in the hippocampus (b), globus pallidus internus (d), cortex (f), interpeduncular nucleus (h), motor nucleus of the trigeminal nerve (j), and hypoglossal nucleus (l). Data are mean ± S.D., n = 3 rodents.

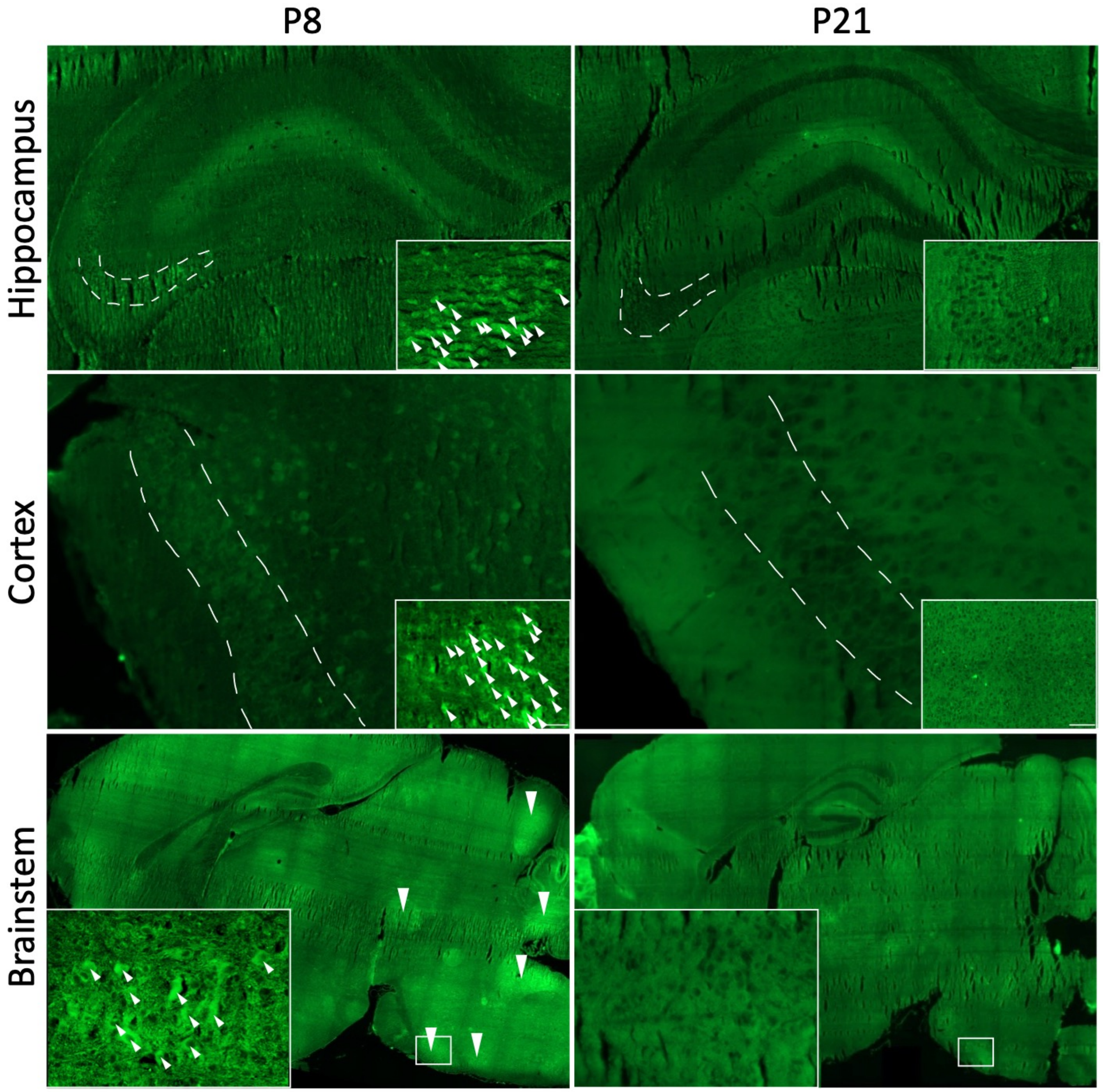

Supplementary Figure 6. CSF interactions with functional-anatomic cell groupings are decreased in P21 mice compared to P8 mice. Hippocampus, cortex, and brainstem cellular uptake of CellTracker CMFDA (green) is observed at P8, but not at P21 (white dashed lines). Arrowheads indicate parenchymal cells with intracellular CellTracker at P8. No CellTracker uptake is present in the corresponding locations at P21 (indicated by dark circular bodies).

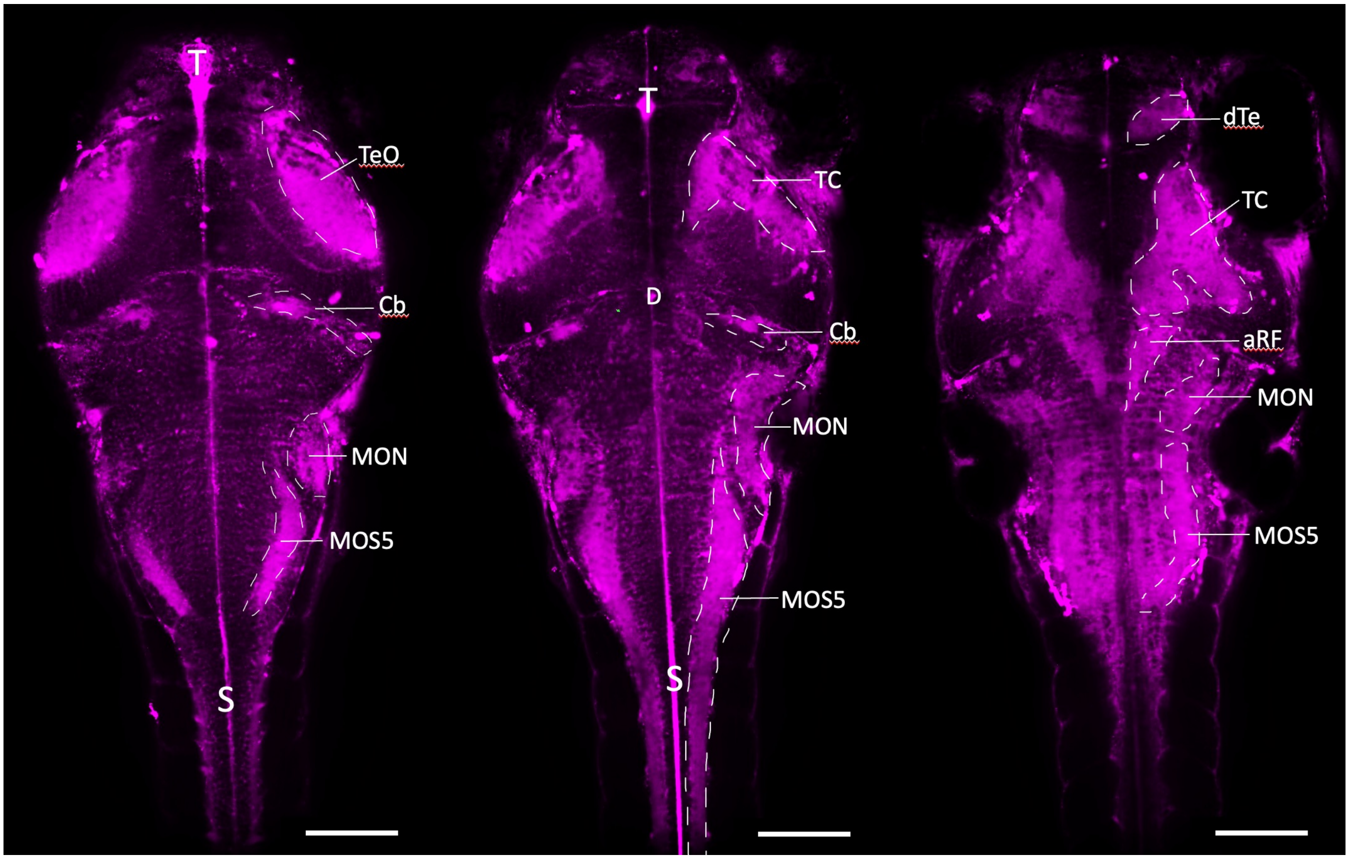

Supplementary Figure 7. Fluorescent CSF tracers preferentially distribute to neuron-rich populations in zebrafish. 70 kDa Red Dextran (magenta) distribution through the zebrafish brain and spinal cord 90 minutes post-intraventricular injection in a 96 hours post-fertilization zebrafish. Dextran is observed in the CSF spaces of the telencephalic ventricle (T), diencephalic ventricle (D), spinal canal (S), and areas of the parenchyma including the optic tectum (TeO), tectum (TC), cerebellum (Cb), medial octavalis nucleus (MON), inferior dorsal medulla oblongata stripe 5 (MOS5), dorsal telencephalon (dTe), and anterior reticular formation (aRF). scalebar = 1 mm. All data are representative of 3 zebrafish.

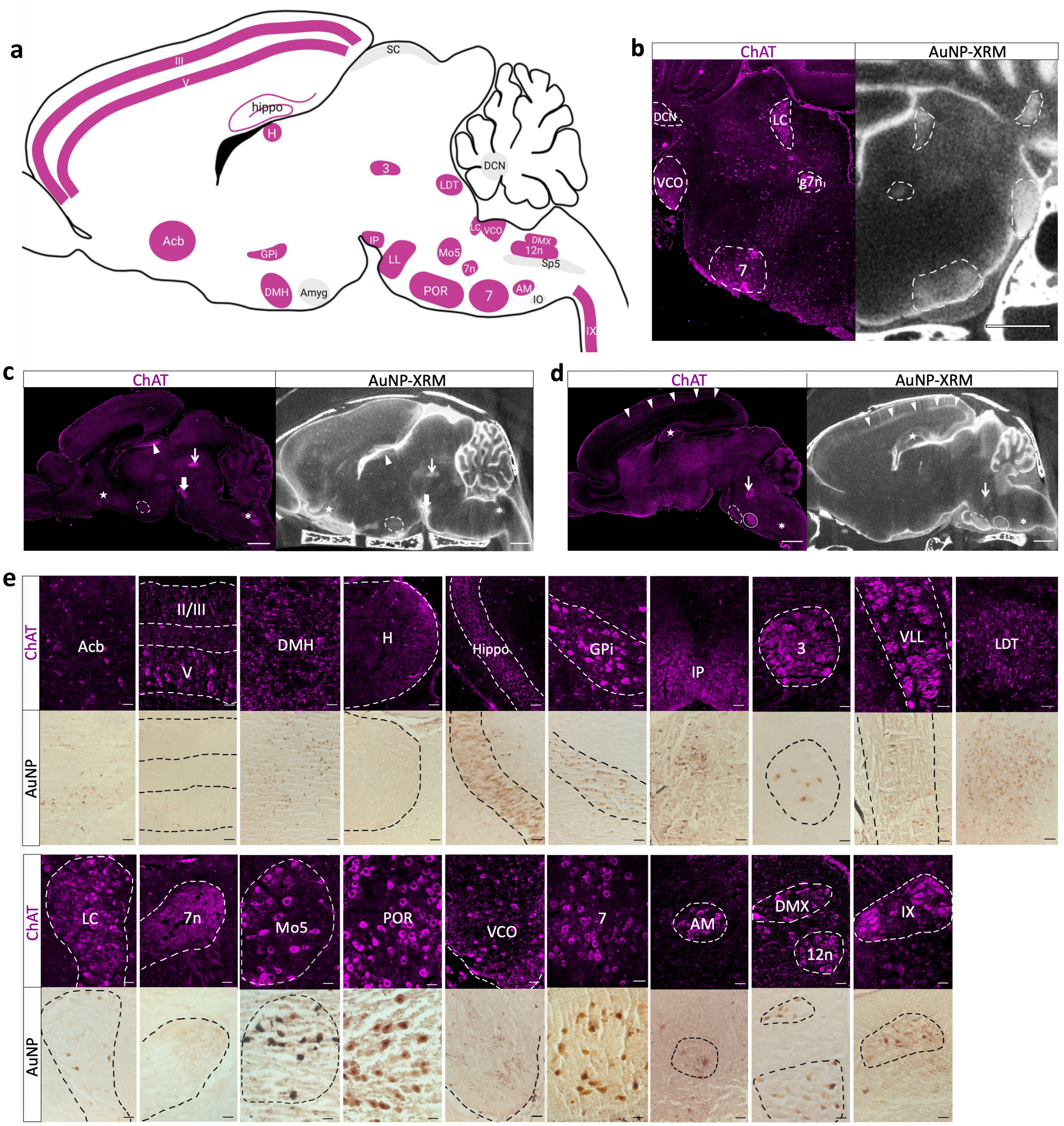

Supplementary Figure 8. CSF distribution mirrors choline acetyltransferase (ChAT) expression in the rodent brain. a, Schematic representation of ChAT-expressing functional-anatomic cell groupings in which 1.9-nm gold nanoparticles (AuNPs) were observed 10 minutes after intraventricular injection at P7 in an aCSF control rodent. Five nuclei with targeted 1.9-nm AuNP interactions, the amygdala (amyg), superior colliculus (SC), spinal trigeminal nucleus (Sp5), inferior olive (IO), and deep cerebellar nuclei (DCN), do not express ChAT. Abbreviations: nucleus accumbens (Acb), neocortex (II/III, V), dorsal medial hypothalamus (DMH), habenula (H), hippocampus (hippo), globus pallidus internus (GPi), amygdala (Amyg), interpenduncular nucleus (IP), oculomotor nucleus (3), lateral lemniscus (LL); locus coeruleus (LC), genu of the facial nerve (g7n), motor trigeminal nucleus (Mo5), periolivary region (POR), ventral cochlear nucleus (VCO), dentate nucleus (DN), facial nerve nucleus (7), nucleus ambiguus (AM), dorsal motor nucleus of the vagus nerve (DMX), hypoglossal nucleus (12n), lamina IX of the spinal cord (IX). **b-e**, Representative coronal (b), sagittal (c-d), and higher magnification (e) immunofluorescence sections showing ChAT expression (left) and AuNP distribution on XRM/histology (right, bottom). c: star, Acb; dotted circle, DMH; arrowhead, H; arrow, 3; asterisk, AM. D: arrowheads, neocortex; star, hippo; arrow, Mo5; dotted circle, POR; circle, 7. b-d scalebars = 1 mm. e scalebars = 50 μm. Data is representative of 3 rodents and all abbreviations are indicated in Supplementary Table 1.

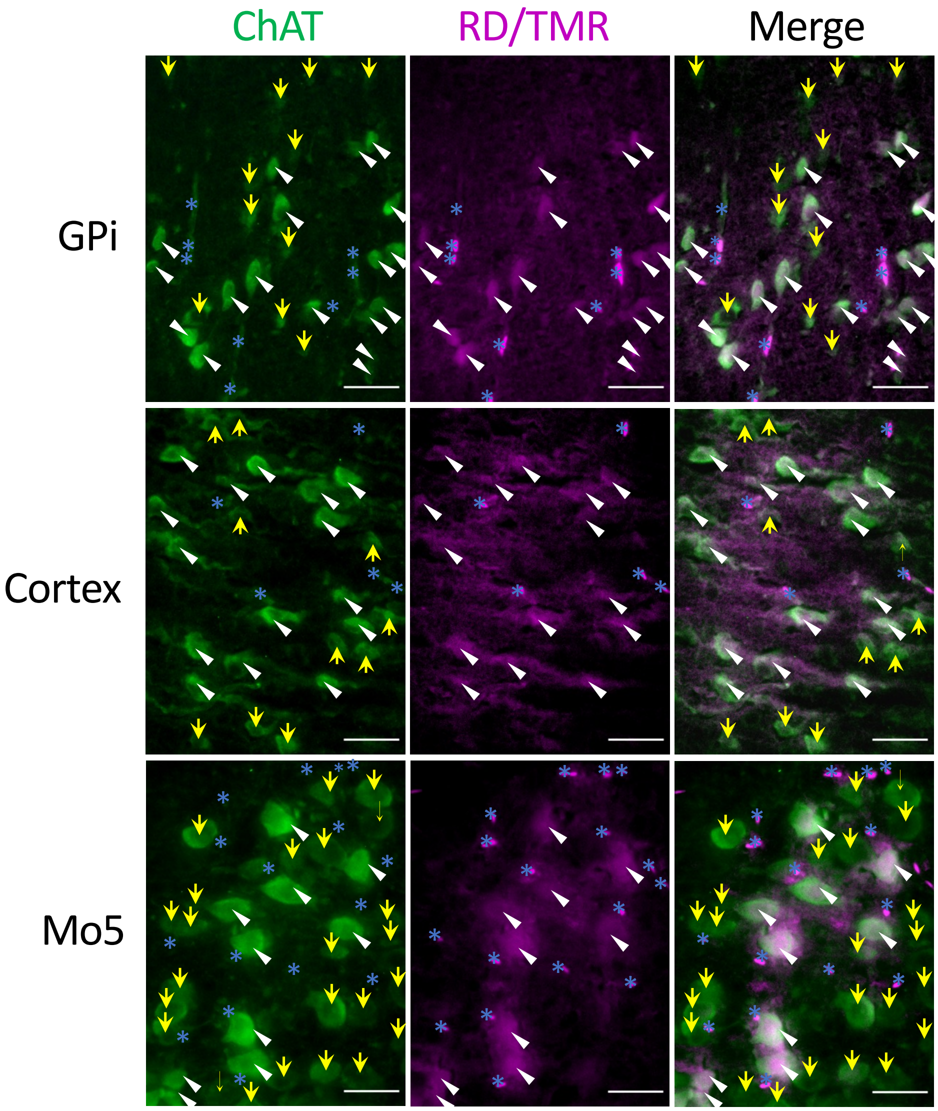

Supplementary Figure 9. Cells within the globus pallidus internus, cortex, and motor nucleus of the trigeminal nerve that interact with CSF tracers express choline acetyltransferase (ChAT). Representative immunofluorescence of Red Dextran/Tetramethylrhodamine (RD/TMR) tracer uptake and ChAT positivity in the globus pallidus internus (GPi), cortex, and motor nucleus of the trigeminal nerve (Mo5). 100% of neurons from these three regions that take up RD/TMR express ChAT (white arrowheads), but not all ChAT-expressing neurons take up RD/TMR (yellow arrowheads). RD/TMR+/ChAT+ cells are indicated with white arrowheads; RD/TMR-/ChAT+ cells are indicated with yellow arrows; blood vessels are indicated with blue asterisks. Data is representative of 3 rodents. Scalebar = 50 μm.

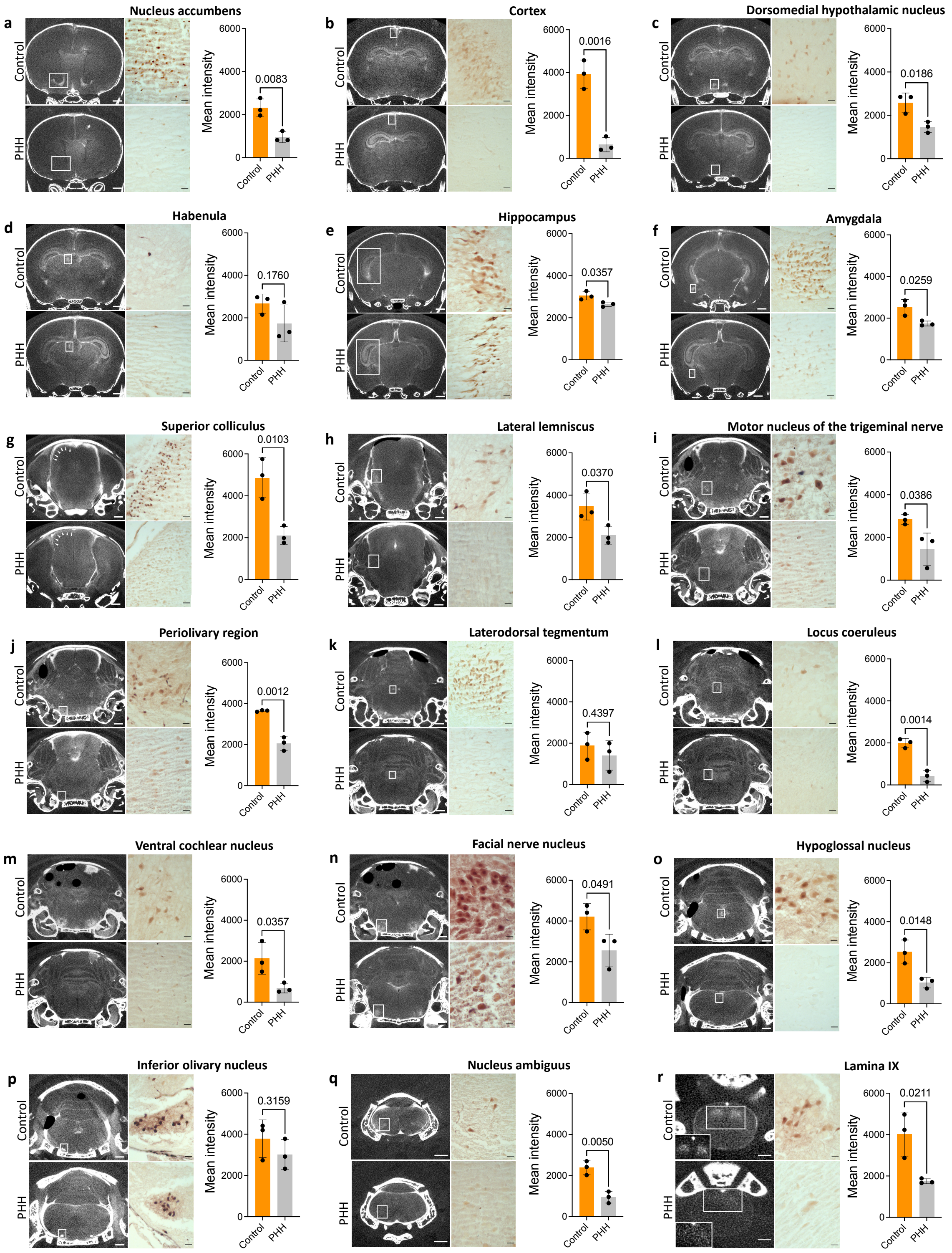

Supplementary Figure 10. Decreased small CSF solute distribution in the brain and spinal cord in posthemorrhagic hydrocephalus (PHH). a, Representative coronal X-ray microtomography (XRM), histology, and quantification showing decreased 1.9-nm AuNP distribution in the nucleus accumbens (a), cortex (b), dorsal medial hypothalamus (c), habenula (d), hippocampus (e), amygdala (f), superior colliculus (g), lateral lemniscus (h), motor nucleus of the trigeminal nerve (i), periolivary region (j), laterodorsal tegmentum (k), locus coeruleus (l), ventral cochlear nucleus (m), facial nerve nucleus (n), hypoglossal nucleus (o), inferior olivary nucleus (p), nucleus ambiguus (q), and lamina IX of the spinal cord (r) in P7 PHH rodents 10 minutes following intraventricular injection compared to aCSF controls. scalebars = 1 mm, histology scalebars = 25 μm. Data are mean ± S.D., n = 3 rodents, unpaired two-tailed t-test. A subset of control XRMs are the same images that appear in Supplementary Figure 1, and Supplementary figure 10o uses the same XRM and histology images that appear in Main Figures 3e and 3f.

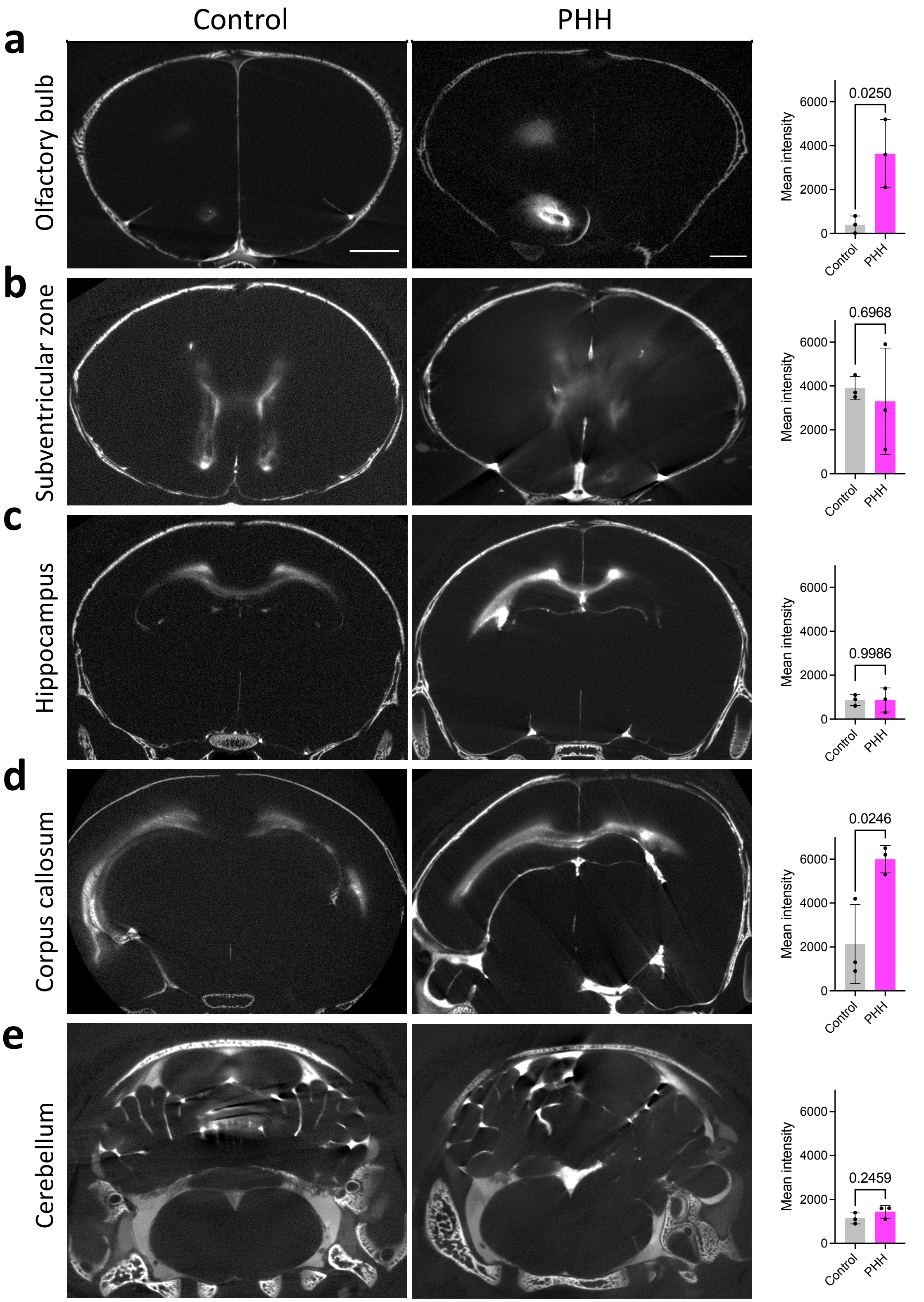

Supplementary Figure 11. Altered distribution of large CSF solutes in the brain and spinal cord parenchyma in posthemorrhagic hydrocephalus (PHH). a-e, Representative coronal x-ray microtomography (XRM) images and quantification of 15-nm AuNP distribution to the olfactory bulb (a), subventricular zone (b), hippocampus (c), corpus callosum (d), and cerebellum (e). Data are mean ± S.D., n = 3 rodents, unpaired two-tailed t-test.

	Region	Abbreviation	P7 ICV 1.9-nm AuNP (rat)	P7 ICM 1.9-nm AuNP (rat)	P7 ICM RD/TMR (rat)	P8 ICM CellTracker (mouse)	P21 ICM CellTracker (mouse)	P7 ICV 15-nm AuNP (rat)
Present in all rats with P7 ICV 1.9-nm AuNP injection								
	Nucleus accumbens	Acb	+	+	+	+	+	-
	Layers III and V of the cortex	III and V	+	+	+	Not evaluated	-	-
	Dorsomedial hypothalamus	DMH	+	+	+	+	Not evaluated	-
	Habenula	H	+	-	-	+	Not evaluated	+ (non-neuronal)
	Hippocampus	hippo	+	+	+	+	-	+ (non-neuronal)
	Globus pallidus internus	GPi	+	Not evaluated	+	+	-	-
	Amygdala	Amyg	+	+	Not evaluated	Not evaluated	Not evaluated	-
	Interpeduncular nucleus	IP	+	+	+	+	-	-
	Superior colliculus	SC	+	+	+	+	+	-
	Oculomotor nucleus	3	+	Not evaluated	+	+	-	-
	Lateral lemniscus	LL	+	+	+	+	Not evaluated	-
	Motor nucleus of the trigeminal nerve	Mo5	+	+	+	+	-	-
	Periolivary region of the superior olivary complex	POR	+	+	+	+	-	-
	Laterodorsal tegmentum	LDT	+	-	+	+	Not evaluated	-
	Locus coeruleus	LC	+	+	+	+	Not evaluated	-
	Ventral cochlear nucleus	VCO	+	+	+	+	Not evaluated	-
	Facial nerve nucleus	7, 7n	+	+	+	+	-	-
	Dorsal motor nucleus of the vagus nerve	DMX	+	+	+	+	Not evaluated	-
	Hypoglossal nucleus	12n	+	+	+	+	Not evaluated	-
	Spinal trigeminal nucleus	Sp5	+	+	+	+	Not evaluated	-

	Inferior olivary nucleus	IO	+	-	-	Not evaluated	Not evaluated	-
	Deep cerebellar nuclei	DCN	+	+	+	+	-	-
	Nucleus ambiguus	AM	+	+	+	+	Not evaluated	-
	Lamina IX of the spinal cord	IX	+	-	-	-	-	-
Present in subset of rats with ICV 1.9-nm AuNP injection								-
	Nucleus of Darkschewitsch	Dk	+ (1/3)	Not evaluated	+	+	Not evaluated	-
	Red nucleus	RN	+ (1/3)	Not evaluated	+	+	Not evaluated	-
	Trochlear nucleus	4	+ (1/3)	Not evaluated	+	+	Not evaluated	-
	Nucleus of the trapezoid body	NTB	+ (1/3)	Not evaluated	Not evaluated	Not evaluated	Not evaluated	-
	Nucleus sagulum	SAG	+ (2/3)	+	Not evaluated	Not evaluated	Not evaluated	-
	External cuneate nucleus	CU	+ (2/3)	+	+	+	Not evaluated	-
	Inferior colliculus	IC	+ (2/3)	+	+	+	+	-
	Parabigeminal nucleus	PBG	+ (2/3)	+	+	+	Not evaluated	-
	Pontine reticular nucleus	N/A	+ (2/3)	+	+	+	Not evaluated	-

Supplementary Table 1. Neuroanatomical abbreviations and presence (+) or absence -) of functional-anatomic nuclei identified with intraventricular injected gold nanoparticle-enhanced X-ray microtomography in P7 rats. Additional abbreviations: ICV, intraventricular; ICM, intra-cisterna magna; AuNP, gold nanoparticle; RD/TMR, Red-Dextran/Tetramethyl rhodamine. + indicates presence of tracer within functional-anatomic region across n = 3 rodents unless specified.

Region	ChAT+ cell bodies or fibers	Description	Reference
Nucleus accumbens	+	Observed on IF (Extended Data Figure 8) shown in previous studies.	[1,2]
Neocortex	+	Observed on IF (Extended Data Figure 8), shown in previous studies.	[3–5]
Hippocampus	+	Observed on IF (Extended Data Figure 8), shown in previous studies.	[6–9]
Habenula	+	Observed on IF (Extended Data Figure 8), shown in previous studies.	[3,10]
Globus pallidus internus	+	Observed on IF (Extended Data Figure 8), shown in previous studies.	[1]
Dorsal medial hypothalamus	+	Observed on IF (Extended Data Figure 8), shown in previous studies.	[1]
Amygdala	0	Not observed on IF. However, previous studies have implicated ChAT expression in the amygdala.	[11–13]
Oculomotor nucleus	+	Observed on IF (Extended Data Figure 8), shown in previous studies.	[1,114]
Superior colliculus	0	Not observed on IF.	
Interpeduncular nucleus	+	Observed on IF (Extended Data Figure 8), shown in previous studies.	[10,15]
Lateral lemniscus	+	Observed on IF (Extended Data Figure 8), shown in previous studies.	[16,17]
Periolivary region	+	Observed on IF (Extended Data Figure 8), shown in previous studies.	[18]
Laterodorsal tegmentum	+	Observed on IF (Extended Data Figure 8), shown in previous studies.	[1]
Locus coeruleus	+	Observed on IF (Extended Data Figure 8)	[19,20]
Ventral cochlear nucleus	+	Observed on IF (Extended Data Figure 8), shown in previous studies.	[21]
Motor trigeminal nucleus	+	Observed on IF (Extended Data Figure 8), shown in previous studies.	[1,2]
Spinal trigeminal nucleus	0	Not observed on IF.	
Facial nerve nucleus	+	Observed on IF (Extended Data Figure 8), shown in previous studies.	[1,10]
Nucleus ambiguus	+	Observed on IF (Extended Data Figure 8), shown in previous studies.	[1]
Dorsal motor vagus nucleus	+	Observed on IF (Extended Data Figure 8), shown in previous studies.	[1]
Hypoglossal nucleus	+	Observed on IF (Extended Data Figure 8), shown in previous studies.	[1,10]
Inferior olivary nucleus	0	Not observed on IF.	
Deep cerebellar nuclei	0	Not observed on IF. Previous studies suggest ChAT positivity in the cerebellar cortex, but not the nuclei.	[22–25]
Lamina IX	+	Observed on IF (Extended Data Figure 8), shown in previous studies.	[1,26]

Supplementary Table 2. ChAT positivity by nuclei and cell groupings with observed 1.9 nm AuNP distribution.

Region	Reference
Caudate/Putamen	[2]
Nucleus basalis of Meynert	[3]
Magnocellular preoptic nucleus	[2]
Olfactory tubercle	[2]
Islands of Calleja	[2]
Nucleus of the diagonal band	[2]
Substantia innominate	[2]
Medial septal nucleus	[2]
Septofimbrial nucleus	[2]
Bed nucleus of the stria terminalis	[2]
Septo-olfactory area	[2]
Edinger Westphal nucleus	[2]
Pedunculopontine tegmental nucleus	[2]
Superior cerebellar peduncle	[2]
Parabrachial nucleus	[2]
Abducens nucleus	[2]

Supplementary Table 3. Subset of known ChAT-positive nuclei and cell groupings with no observed 1.9 nm AuNP distribution.

References

1. Armstrong, D. M., Saper, C. B., Levey, A. I., Wainer, B. H. & Terry, R. D. Distribution of cholinergic neurons in rat brain: Demonstrated by the immunocytochemical localization of choline acetyltransferase. *Journal of Comparative Neurology* **216**, (1983).

2. GENSAT Project at Rockefeller University, Mouse Brain Atlas, Image Navigator. http://www.gensat.org/imagenavigator.jsp?imageID=29461.

3. Oda, Y. & Nakanishi, I. The distribution of cholinergic neurons in the human central nervous system. *Histology and histopathology* **15**, 825–834 (2000).

4. Granger, A. J. *et al.* Cortical ChAT+ neurons co-transmit acetylcholine and GABA in a target-and brain-region-specific manner. *eLife* **9**, 1–29 (2020).

5. Houser, C. R., Crawford, G. D., Salvaterra, P. M. & Vaughn, J. E. Immunocytochemical localization of choline acetyltransferase in rat cerebral cortex: a study of cholinergic neurons and synapses. *The Journal of comparative neurology* **234**, 17–34 (1985).

6. Hawley, W. R., Witty, C. F., Daniel, J. M. & Dohanich, G. P. Choline acetyltransferase in the hippocampus is associated with learning strategy preference in adult male rats. *Behavioural brain research* **289**, 118–124 (2015).

7. Meredith, G. E. & Wouterlood, F. G. Hippocampal and midline thalamic fibers and terminals in relation to the choline acetyltransferase-immunoreactive neurons in nucleus accumbens of the rat: A light and electron microscopic study. *Journal of Comparative Neurology* **296**, 204–221 (1990).

8. Frotscher, M. & Léránth, C. Cholinergic innervation of the rat hippocampus as revealed by choline acetyltransferase immunocytochemistry: a combined light and electron microscopic study. *The Journal of comparative neurology* **239**, 237–246 (1985).

9. GENSAT Project at Rockefeller University, Mouse Brain Atlas, Image Navigator. http://www.gensat.org/imagenavigator.jsp?imageID=29460.

10. GENSAT Project at Rockefeller University, Mouse Brain Atlas, Image Navigator. http://www.gensat.org/imagenavigator.jsp?imageID=29464.

11. Hellendall, R. P., Godfrey, D. A., Ross, C. D., Armstrong, D. M. & Price, J. L. The distribution of choline acetyltransferase in the rat amygdaloid complex and adjacent cortical areas, as determined by quantitative micro-assay and immunohistochemistry. *The Journal of comparative neurology* **249**, 486–498 (1986).

12. Emson, P. C., Paxinos, G., le Gal La Salle, G., Ben-Ari, Y. & Silver, A. Choline acetyltransferase and acetylcholinesterase containing projections from the basal forebrain to the amygdaloid complex of the rat. *Brain research* **165**, 271–282 (1979).

13. Ben-Ari, Y., Zigmond, R. E., Shute, C. C. D. & Lewis, P. R. Regional distribution of choline acetyltransferase and acetylcholinesterase within the amygdaloid complex and stria terminalis system. *Brain research* **120**, 435–445 (1977).

14. GENSAT Project at Rockefeller University, Mouse Brain Atlas, Image Navigator. http://www.gensat.org/imagenavigator.jsp?imageID=29463.

15. Lenn, N. J., Leranth, C. & Zaborszky, L. Choline acetyltransferase immunoreactivity is localized to four types of synapses in the rat interpeduncular nucleus. *Journal of neurocytology* **14**, 909–919 (1985).

16. Motts, S. D. & Schofield, B. R. Sources of Cholinergic Input to the Inferior Colliculus. *Neuroscience* **160**, 103 (2009).

17. Lauterborn, J. C., Isackson, P. J., Montalvo, R. & Gall, C. M. In situ hybridization localization of choline acetyltransferase mRNA in adult rat brain and spinal cord. *Brain research. Molecular brain research* **17**, 59–69 (1993).

18. Simmons, D. D., Bertolotto, C., Typpo, K., Clay, A. & Wu, M. Differential development of cholinergic-like neurons in the superior olive: a light microscopic study. *Anatomy and embryology* **200**, 585–595 (1999).

19. Shiromani, P. J., Armstrong, D. M., Berkowitz, A., Jeste, D. v & Gillin, J. C. Distribution of Choline Acetyltransferase Immunoreactive Somata in the Feline Brainstem: Implications for REM Sleep Generation. *Sleep* **11**, 1–16 (1988).

20. Strong, R. *et al.* Degeneration of the cholinergic innervation of the locus ceruleus in Alzheimer's disease. *Brain research* **542**, 23–28 (1991).

21. Henderson, Z. & Sherriff, F. E. Distribution of choline acetyltransferase immunoreactive axons and terminals in the rat and ferret brainstem. *The Journal of comparative neurology* **314**, 147–163 (1991).

22. Jaarsma, D. *et al.* Cholinergic innervation and receptors in the cerebellum. *Progress in Brain Research* **114**, 67–96 (1997).

23. Barmack, N. H., Baughman, R. W. & Eckenstein, F. P. Cholinergic innervation of the cerebellum of rat, rabbit, cat, and monkey as revealed by choline acetyltransferase activity and immunohistochemistry. *Journal of Comparative Neurology* **317**, 233–249 (1992).

24. Ojima, H., Kawajiri, S. -I & Yamasaki, T. Cholinergic innervation of the rat cerebellum: Qualitative and quantitative analyses of elements immunoreactive to a monoclonal antibody against choline acetyltransferase. *Journal of Comparative Neurology* **290**, 41–52 (1989).

25. de Lacalle, S., Hersh, L. B. & Saper, C. B. Cholinergic innervation of the human cerebellum. *The Journal of comparative neurology* **328**, 364–376 (1993).

26. GENSAT Project at Rockefeller University, Mouse Brain Atlas, Image Navigator. http://www.gensat.org/imagenavigator.jsp?imageID=29467.
