# Supplementary material for: Gold nanoparticle-enhanced X-ray microtomography of the rodent reveals region-specific cerebrospinal fluid circulation in the brain
Source: Nat Commun. 2023 Jan 27;14:453. doi: 10.1038/s41467-023-36083-1 (PMC9883388; doi:10.1038/s41467-023-36083-1)
Supplement: Supplementary file 3 — Reporting Summary [file 41467_2023_36083_MOESM3_ESM.pdf]

nature portfolio

Corresponding author(s): Jennifer M Strahle, MD

Last updated by author(s): December 14, 2022

Reporting Summary

Nature Portfolio wishes to improve the reproducibility of the work that we publish. This form provides structure for consistency and transparency in reporting. For further information on Nature Portfolio policies, see our Editorial Policies and the Editorial Policy Checklist.

Statistics

For all statistical analyses, confirm that the following items are present in the figure legend, table legend, main text, or Methods section.

n/a	Confirmed	
☐	☒	The exact sample size (*n*) for each experimental group/condition, given as a discrete number and unit of measurement
☐	☒	A statement on whether measurements were taken from distinct samples or whether the same sample was measured repeatedly
☐	☒	The statistical test(s) used AND whether they are one- or two-sided<br>*Only common tests should be described solely by name; describe more complex techniques in the Methods section.*
☐	☒	A description of all covariates tested
☐	☒	A description of any assumptions or corrections, such as tests of normality and adjustment for multiple comparisons
☐	☒	A full description of the statistical parameters including central tendency (e.g. means) or other basic estimates (e.g. regression coefficient) AND variation (e.g. standard deviation) or associated estimates of uncertainty (e.g. confidence intervals)
☐	☒	For null hypothesis testing, the test statistic (e.g. *F*, *t*, *r*) with confidence intervals, effect sizes, degrees of freedom and *P* value noted<br>*Give P values as exact values whenever suitable.*
☒	☐	For Bayesian analysis, information on the choice of priors and Markov chain Monte Carlo settings
☒	☐	For hierarchical and complex designs, identification of the appropriate level for tests and full reporting of outcomes
☒	☐	Estimates of effect sizes (e.g. Cohen's *d*, Pearson's *r*), indicating how they were calculated

Our web collection on statistics for biologists contains articles on many of the points above.

Software and code

Policy information about availability of computer code

Data collection	The following microscopes/imaging devices/software were used to collect the data in this study:<br>- Zeiss Xradia Versa 520 X-ray microscope<br>- Zeiss 880 Two-Photon microscope (inverted) with 488 and 561 laser lines and 20x (dry), 40x (oil immersion), and 63x (oil immersion) lenses.<br>- Bruker 9.4-T (400-MHz), 20-cm clear bore MRI scanner with a mouse brain CryoProbe<br>- Union BioMetrica VAST BioImager Platform<br>- Zeiss XM3DViewer 1.2.8<br>- Dragonfly 2020.1<br>- Paravision 360 3.2

Data analysis	The following softwares were used to analyze the data in this study:<br>- FIJI image processing software (NIH) version 2.3.0/1.53q<br>- ITK-SNAP 4.0.0 beta<br>- GraphPad Prism v9.0.0<br>- Microsoft Office Excel Version 16.36<br>- Connectome Workbench v1.5.0<br>- 4dfp tools (http://4dfp.readthedocs.io)<br>- mapZebrain zebrafish atlas (https://mapzebrain.org/atlas/2d)<br>- Custom code used for registration and skull stripping is available in the Supplementary Information

For manuscripts utilizing custom algorithms or software that are central to the research but not yet described in published literature, software must be made available to editors and reviewers. We strongly encourage code deposition in a community repository (e.g. GitHub). See the Nature Portfolio guidelines for submitting code & software for further information.

Data

Policy information about availability of data

All manuscripts must include a data availability statement. This statement should provide the following information, where applicable:

- Accession codes, unique identifiers, or web links for publicly available datasets
- A description of any restrictions on data availability
- For clinical datasets or third party data, please ensure that the statement adheres to our policy

The datasets generated and/or analyzed during the current study are available in the Supplementary Data 1 file linked in the Supplementary Information section. XRM and MRI images are not publicly available due to their size, but are available from the corresponding author on reasonable request.

Human research participants

Policy information about studies involving human research participants and Sex and Gender in Research.

Reporting on sex and gender	No human research participants were included in this study.
Population characteristics	No human research participants were included in this study.
Recruitment	No human research participants were included in this study.
Ethics oversight	No human research participants were included in this study.

Note that full information on the approval of the study protocol must also be provided in the manuscript.

Field-specific reporting

Please select the one below that is the best fit for your research. If you are not sure, read the appropriate sections before making your selection.

- [x] Life sciences
- [ ] Behavioural & social sciences
- [ ] Ecological, evolutionary & environmental sciences

For a reference copy of the document with all sections, see nature.com/documents/nr-reporting-summary-flat.pdf

Life sciences study design

All studies must disclose on these points even when the disclosure is negative.

Sample size	Statistical methods were not used to re-calculate or predetermine sample sizes in any experiments. Sample size was determined based on similar studies with XRM/MicroCT and is consistent with recently published studies utilizing XRM/MicroCT (Maes et al, Nature Communications, October 2022). Since this study introduced a novel use of XRM to imaging CSF pathways, we primarily aimed to show the applicability and repeatability of this technique to tracking CSF, for which we deemed n = 3 for each condition was sufficient for proof-of concept. Our findings with XRM were also confirmed with additional histological analyses.
Data exclusions	No data were excluded from the analyses.
Replication	To ensure reproducibility, all XRM data presented in this manuscript were repeated three times. All fluorescent tracer injections, immunofluorescence, MRI and histological experiments were repeated at least three times. All confocal and histological images presented were imaged at least three times for a single data point. Results for all technical and biological replicates were consistent among their groups. The exact number of replications of each experiment is stated in the corresponding Figure Legend.
Randomization	All samples were randomly allocated into experimental groups by randomly choosing pups from each litter to receive different treatments. Rats used in this study were randomly chosen from their litters to receive artificial CSF or hemoglobin injections to create the control and PHH conditions. Rats were also randomly chosen to receive 1.9 nm or 15 nm gold nanoparticle injection or Red Dextran injection. Rats were also

	randomly chosen to receive intracisterna magna vs. intraventricular injection. Mice and zebrafish used in this study were also randomly chosen to receive tracer injections.
Blinding	Blinding was not performed for MRI image acquisition because imaging occurred shortly following the artificial CSF or hemoglobin injection, so the same experimenter performed injection and MRI acquisition. Experimental conditions were also evident from the MRI image data. Blinding was performed for all other parts of this study, including XRM image acquisition and post-processing, gold nanoparticle and other tracer injection, immunostaining, histology, tissue harvesting, all quantifications and analyses.

Reporting for specific materials, systems and methods

We require information from authors about some types of materials, experimental systems and methods used in many studies. Here, indicate whether each material, system or method listed is relevant to your study. If you are not sure if a list item applies to your research, read the appropriate section before selecting a response.

Materials & experimental systems

n/a	Involved in the study	
☐	☒	Antibodies
☒	☐	Eukaryotic cell lines
☒	☐	Palaeontology and archaeology
☐	☒	Animals and other organisms
☒	☐	Clinical data
☒	☐	Dual use research of concern

Methods

n/a	Involved in the study	
☒	☐	ChIP-seq
☒	☐	Flow cytometry
☐	☒	MRI-based neuroimaging

Antibodies

Antibodies used	The following primary antibodies were used for IHC on formalin-fixed paraffin-embedded tissues:<br>1. Rabbit monocolonal [EPR16590], anti-ChAT, Abcam, Cat# ab178850, 1:200 dilution, LOT: GR3230471-7<br>2. Rabbit monoclonal [EP1576Y], anti-S100 beta, Abcam, Cat# ab52642, 1:200 dilution, LOT: GR3215095-2<br>3. Mouse monoclonal, anti-GFAP-Cy3, Sigma-Aldrich, Cat# C9205, 1:200 dilution, LOT: 0000182455<br>4. Rabbit monoclonal [EPR12763], anti-NeuN, Abcam, Cat# 177487, 1:200 dilution, LOT: GR249899-42<br><br>The following secondary antibodies were used for IHC in formalin-fixed paraffin-embedded tissues:<br>5. Goat anti-Rabbit IgG (H+L) Cross-Absorbed Secondary Antibody, Alexa Fluor 594, Invitrogen, Cat# A-32740, 1:2000 dilution, LOT: 2201598<br>6. Goat anti-Rabbit IgG (H+L) Cross-Absorbed Secondary Antibody, Alexa Fluor 488, Invitrogen, Cat# A-11008, 1:2000 dilution, LOT: 2105157
Validation	Each antibody was validated by the manufacturer. The manufacturers for the corresponding antibodies are listed below:<br>1. https://www.abcam.com/choline-acetyltransferase-antibody-epr16590-ab178850.html#description_references<br>2. https://www.abcam.com/s100-beta-antibody-ep1576y-astrocyte-marker-ab52642.html#description_references<br>3. https://www.sigmaaldrich.com/US/en/product/sigma/c9205?gclid=CjwKCAiAheacBhB8EiwAItVO21L2p2j6lf3osdOCSE2tbfNq3ekHCCQEbpj60H5MMB_EPcGxjlrHvhoC9W8QAvD_BwE&gclsrc=aw.ds<br>4. https://www.abcam.com/neun-antibody-epr12763-neuronal-marker-ab177487.html<br>5. https://www.thermofisher.com/antibody/product/Goat-anti-Rabbit-IgG-H-L-Highly-Cross-Adsorbed-Secondary-Antibody-Polyclonal/A32740<br>6. https://www.thermofisher.com/antibody/product/Goat-anti-Rabbit-IgG-H-L-Cross-Adsorbed-Secondary-Antibody-Polyclonal/A-11008<br><br>The usage of these antibodies is described in full in the methods. Sections were soaked in xylene to remove paraffin, before hydration through descending grades of alcohol (100%, 95%, 70%, 50%, 30%) to DDW. Antigen retrieval was performed using a pH 6.0 citrate buffer (Sigma-Aldrich, St. Louis, MO) by microwaving in a 1:1000 solution in DDW for 30 minutes. Slides were cooled to room temperature, rinsed with PBS and blocked in 5% normal goat serum, 2.5% BSA, 0.5% TX-100 in PBS for one hour. Sections were incubated with appropriate dilutions of primary antibodies in PBS with 1% BSA and 0.5% TX-100 (TBST) overnight at 4°C. Sections were then rinsed in PBS 6 times for 5 minutes each followed by incubation for 90 minutes at room temperature with secondary antibody diluted in TBST. Sections were then rinsed in PBS 5-6 times for 5 minutes each at room temperature followed by incubation with DAPI reagent in a 1:500 dilution. After 5 minutes in DAPI, sections were washed in PBS 3 times for 5 minutes and mounted.

Animals and other research organisms

Policy information about studies involving animals; ARRIVE guidelines recommended for reporting animal research, and Sex and Gender in Research

Laboratory animals	Sprague Dawley Rats (Charles River Laboratories, Wilmington, MA) and C57BL/6J mice (The Jackson Laboratory, Bar Harbor, ME) were housed in a 12-hour light-dark cycle in a temperature and humidity-controlled room. Water and food were provided ad libitum. Rat and mouse pups were obtained from Charles River/Jackson Laboratories with their dams and housed at the animal facility with

	their dams until P21. Zebrafish were bred in house.<br><br>Species: Rat; Strain: Sprague Dawley (Charles River Laboratories); Sex: male and female rats; Age: post-natal day 4-7.<br>Species: Mouse; Strain C57BL/6J (The Jackson Laboratory); Sex: male and female mice; Age: post-natal day 7-8.<br>Species: Zebrafish; Strain Casper line crossed to mitfa-/-Tg(HuC:EGFP); Sex: male and female; Age: 4-5 DPF.
Wild animals	This study did not involve wild animals.
Reporting on sex	Sex was not selected for over the duration of this study. Sex data was not collected for the XRM scans and as a result sex-based analyses were not conducted.
Field-collected samples	This study did not involve samples collected from the field.
Ethics oversight	All experiments were approved by the Washington University in St. Louis Institutional Animal Care and Use Committee (protocols #19-0905 and 19-1026).

Note that full information on the approval of the study protocol must also be provided in the manuscript.

Magnetic resonance imaging

Experimental design

Design type	1. Intraventricular gadolinium contrast (gadoterate meglumine, Gd-DOTA) to trace cerebrospinal fluid circulation<br>2. Structural T2-weighted fast spin echo MRI to assess ventricle volumes 72 hours following aCSF or hemoglobin injection into the lateral ventricles
Design specifications	1. Each rat received a pre-contrast scan and a post-contrast scan.<br>2. Each rat received one MRI scan. No rats underwent multiple or sequential scans.
Behavioral performance measures	N/A

Acquisition

Imaging type(s)	1. Structural T1-weighted imaging<br>2. Structural T2-weighted fast spin echo imaging
Field strength	1. 9.4T<br>2. 4.7T
Sequence & imaging parameters	1. Repetition time 800/echo time 7.8195 mS, 4 averages, field of view 24.0 x 24.0 mm, matrix 256 x 256, 24 axial slices, 0.50mm thick, and 14 sagittal slices, 1.0mm thick.<br>2. Repetition time 3000/echo time 27.50 mS, 3 averages, field of view was 18.0 mm x 18.0 mm, matrix 128 x 128, 24 axial slices, and 0.50 mm thick
Area of acquisition	Whole rat head

Diudiffusion MRI ☐ Used ☒ Not used

Preprocessing

Preprocessing software	Paravision 360 3.2
Normalization	1. Each rat in the received a pre-contrast scan and was its own control.<br>2. N/A
Normalization template	1. Each rat in the received a pre-contrast scan and was its own control.<br>2. N/A
Noise and artifact removal	No noise and artifact removal was performed.
Volume censoring	No volume censoring was performed.

Statistical modeling & inference

Model type and settings	1. Tracer diffusion<br>2. Ventricle size after aCSF or Hb injection
Effect(s) tested	1. N/A<br>2. Unpaired, two-tailed t-test (aCSF ventricle volume vs. Hb ventricle volume)

Specify type of analysis: ☐ Whole brain ☒ ROI-based ☐ Both

Anatomical location(s)	Lateral ventricles
Statistic type for inference (See Eklund et al. 2016)	N/A
Correction	N/A

Models & analysis

n/a	Involved in the study
☒	☐ Functional and/or effective connectivity
☒	☐ Graph analysis
☒	☐ Multivariate modeling or predictive analysis
